# Incidence, mortality, and risk factors of bladder, kidney, prostate and testicular cancers in China and comparisons with the United States, the United Kingdom, Japan, and the Republic of Korea: an up-to-date overview based on the Global Burden of Disease 2021

**DOI:** 10.1186/s40164-025-00694-9

**Published:** 2025-08-06

**Authors:** Gaohaer Kadeerhan, Zhongji Jiang, Hong Guo, Xinzhi Ma, Jin Zhang, Wenmin Guo, Jiedong Jia, Yibo Gao, Dongwen Wang

**Affiliations:** 1 Central Laboratory & Shenzhen Key Laboratory of Epigenetics and Precision Medicine for Cancers, National Cancer Center/National Clinical Research Center for Cancer/Cancer Hospital & Shenzhen Hospital, Chinese Academy of Medical Sciences and Peking Union Medical College, Shenzhen, 518116 China; 2 Department of Urology, National Cancer Center/National Clinical Research Center for Cancer/Cancer Hospital & Shenzhen Hospital, Chinese Academy of Medical Sciences and Peking Union Medical College, Shenzhen, 518116 China; 3 School of Medicine, Southern University of Science and Technology, Shenzhen, Guangdong 518055 China; 4 First College of Clinical Medicine, Shanxi Medical University, Taiyuan, Shanxi 030001 China; 5 Department of Urology, First Hospital of Shanxi Medical University, Taiyuan, Shanxi 030001 China; 6 Department of Thoracic Surgery, National Cancer Center/National Clinical Research Center for Cancer/Cancer Hospital, Chinese Academy of Medical Sciences and Peking Union Medical College, Beijing, 100021 China; 7 Laboratory of Translational Medicine, National Cancer Center/National Clinical Research Center for Cancer/Cancer Hospital, Chinese Academy of Medical Sciences and Peking Union Medical College, Beijing, 100021 China; 8 State Key Laboratory of Molecular Oncology, National Cancer Center/National Clinical Research Center for Cancer/Cancer Hospital, Chinese Academy of Medical Sciences and Peking Union Medical College, Beijing, 100021 China; 9 Department of Gastroenterology, Shanxi Province Cancer Hospital/Shanxi Hospital Affiliated to Cancer Hospital, Chinese Academy of Medical Sciences/Cancer Hospital Affiliated to Shanxi Medical University, Taiyuan, 030013 China

**Keywords:** Bladder cancer, Kidney cancer, Prostate cancer, Testicular cancer, Incidence, Mortality, Risk factor

## Abstract

**Background:**

The burden of genitourinary cancers has significantly changed in China over the recent decades. This study aims to identify the epidemiological trends and disparities in four common genitourinary cancers, including bladder, kidney, prostate, and testicular cancers, to inform public health strategies and interventions.

**Methods:**

Based on the Global Burden of Disease Study 2021, we examined incident cases, mortality, age-standardized incidence rates (ASIRs), age-standardized mortality rates (ASMRs), mortality-to-incidence ratios (MIRs), and risk factors for four genitourinary cancers globally and in the East Asia and Pacific, China, Japan, the Republic of Korea, the United States, and the United Kingdom from 1990 to 2021 across four specified age groups: 0–14, 15–49, 50–74, and ≥ 75 years. Trend analysis was conducted using Joinpoint analysis to calculate the average annual percentage changes (AAPCs). Decomposition analysis was performed to identify the population-level factors contributing to these trends.

**Results:**

In 2021, China reported approximately 266,887 incident cases and 108,589 deaths from genitourinary cancers, exhibiting distinct age-related patterns. ASIRs for male kidney cancer among those aged 0–14 years and testicular cancer among those aged ≥ 75 years, as well as ASMRs for male bladder cancer aged 15–49 years and testicular cancer aged ≥ 75 years, were higher in China than in the studied regions and countries. The MIRs for genitourinary cancers were generally higher in China. From 1990 to 2021, a notable increase in ASIRs for genitourinary cancers in both sexes, as well as ASMRs for male kidney and prostate cancers, across age groups ranging from 15 to 49 years to ≥ 75 years was observed in China, accompanied by higher AAPCs. The decomposition analysis identified the key population-level contributors to the incidence and mortality trends of genitourinary cancers, highlighting the varying influences of aging, population growth, and epidemiological changes. Smoking-related genitourinary cancer deaths remained high in Chinese males, and mortality related to high body mass index for kidney cancer and elevated fasting plasma glucose levels for bladder cancer also increased.

**Conclusions:**

The distinct age-specific patterns, elevated rates within specific age groups, and marked upward temporal trends of genitourinary cancers in China underscore the critical need for targeted, age-stratified public health interventions.

**Supplementary Information:**

The online version contains supplementary material available at 10.1186/s40164-025-00694-9.

## Introduction

Bladder, kidney, prostate, and testicular cancers are the most common genitourinary cancers worldwide, with a total of 2.59 million new cases and 780,000 deaths in 2022 [1]. Bladder cancer is estimated to be the ninth most frequently diagnosed cancer in both sexes, while prostate cancer is ranked as the second most common cancer and the fifth leading cause of cancer death among men [1]. Although the incidence of kidney and testicular cancers is relatively low, rapidly increasing rates have been noted in recent decades [2–4]. With population growth and aging, the incidence of genitourinary cancers is expected to increase further, which will continue to impose a considerable burden on public health care systems.

Developed areas, such as North America and Europe, report the highest incidence rates of genitourinary cancers. In contrast, Asian countries, despite having lower rates, have experienced a concerning increase [2, 4–6]. This trend is noteworthy in countries with large populations, such as China [7], suggesting that even with lower rates, the actual number of genitourinary cancer cases remains substantially high. Like other malignancies, genitourinary cancers show a progressively rising incidence and mortality with advancing age, posing complex therapeutic and management challenges. Thus, an accurate understanding of the age-related patterns is crucial for enabling targeted prevention and intervention strategies, and for identifying the most vulnerable populations in China. However, research into these age-specific trends remains limited.

Japan and the Republic of Korea, which share racial, genetic, and cultural backgrounds with China, have also experienced an increasing incidence of genitourinary cancers [8, 9]. Considering that they are further along in the stages of population aging and socioeconomic development, their experiences provide important insights for managing genitourinary cancers. Additionally, strategies from highly developed countries such as the United Kingdom (UK) and the United States (US) offer valuable references for global prevention and control efforts. Therefore, comparative studies among countries such as China, the US, the UK, Japan, and the Republic of Korea can inform health care resource allocation and help prioritize the needs of both older and younger populations at higher risk of genitourinary cancers.

In this study, we conducted a comparative analysis of age-stratified (0–14, 15–49, 50–74, and ≥ 75 years) incidence and mortality trends for bladder, kidney, prostate, and testicular cancers across the global, East Asia and Pacific, China, the US, the UK, Japan, and the Republic of Korea, using data from the Global Burden of Disease (GBD) 2021 study. Our research aims to provide policy planning and enhance cancer management approaches for genitourinary cancers.

## Methods

### Data source

Data on bladder, kidney, prostate, and testicular cancers globally, in the East Asia and Pacific region, and in China, Japan, the Republic of Korea, the US, and the UK were obtained from the GBD 2021 study [10]. Briefly, the GBD 2021 study is a comprehensive assessment of global health and provides epidemiological data on 371 diseases and injuries, including genitourinary cancers, for 204 countries and territories from 1990 to 2021. The GBD 2021 study integrated incidence and mortality data from diverse sources, including vital registration, surveys, hospital records, and cancer registries. Advanced statistical modeling was employed for downstream analyses, including meta-regression Bayesian, regularized, trimmed (MR-BRT) methods, DisMod-MR 2.1, and spatiotemporal Gaussian process regression (ST-GPR). Disease classification followed the International Classification of Diseases (ICD) coding standards to ensure data accuracy and cross-study comparability. All analyses complied with the Guidelines for Accurate and Transparent Health Estimates Reporting (GATHER). Further details of the GBD 2021 methodology can be found in previous studies [10–15]. We utilized the Global Health Data Exchange’s query tool (https://ghdx.healthdata.org/gbd-results-tool) to extract the regional and national incidence, deaths, and incidence and mortality rates by age groups of 0–14, 15–49, 50–74 and ≥ 75 years and sex.

### Decomposition analysis

We assessed the relative contributions of three population-level factors: aging, population growth, and epidemiological shifts, to elucidate the drivers of changes in the incidence and mortality of genitourinary cancers from 1990 to 2021. For the decomposition analysis, we employed the Das Gupta method [16], a widely recognized approach in epidemiological and demographic studies [17, 18]. This analysis allowed us to dissect the net contribution of these factors to the observed trends, providing both the absolute changes in incidence and mortality due to each factor and their proportional contributions. Positive proportions indicate that the factor contributed in the same direction as the observed change, whereas negative proportions suggest an opposing effect. For a more detailed explanation of the calculation, see the Supplementary Methods.

### Risk factor analysis

The GBD 2021 study provided a detailed analysis of the impacts of risk factor exposure on particular health outcomes [10]. Data on risk factors for genitourinary cancers were sourced from the GBD 2021, which employed a standardized seven‒step comparative risk assessment (CRA) framework. The analytical process began with quantifying effect sizes through relative risks (RRs) estimation for specific risk‒outcome pairs, applying rigorous causal inference criteria that included consistent evidence from at least two independent epidemiological studies, biological plausibility, dose‒response relationships, and minimal residual confounding after statistical adjustment. Next, age-, sex-, location-, and year-specific exposure distributions were estimated through systematic data synthesis and Bayesian modeling. Subsequent steps involved defining theoretical minimum risk exposure levels (TMRELs) based on epidemiological evidence of risk‒outcome relationships. The population attributable fractions (PAFs) were then computed by comparing the current exposures to the TMRELs using the standard formula:

$$\:PAF\:=\:\:\frac{{\sum\:}_{i=1}^{n}{P}_{i\:}({RR}_{i}-1)}{1+{\sum\:}_{i=1}^{n}{P}_{i\:}({RR}_{i}-1)}$$

where *Pi* = the population proportion exposed to level i, and *RRi* = the relative risk at that level (with the TMREL group *RR* = 1). Additionally, standardized exposure values were generated to enable cross‒risk comparisons. A path analysis was then performed to account for intermediate-risk factors through mediation analysis. The process concluded by calculating the final attributable burdens through PAF‒death integration.

In accordance with the CRA framework, our analysis focused on three modifiable risk factors for genitourinary cancers as established in GBD 2021: smoking as a risk factor for prostate, bladder and kidney cancers [19, 20]; elevated fasting plasma glucose (FPG, any level above 4.9‒5.3 mmol/L) for bladder cancer [21]; and a high body mass index (BMI, greater than 20–23 kg/m^2^) for kidney cancer [22]. RRs for each risk‒cancer pair were obtained from the GBD 2019 [23], with detailed values provided in Table S7. All final PAFs and attributable deaths by age group, sex, location, and year were extracted directly from the GBD Results Tool. The PAF represents the proportion of cancers that could have been prevented if exposure to the risk factor had been reduced to the theoretical minimum risk exposure level [10, 24]. The attributable number, on the other hand, refers to the absolute count of cancer cases directly attributed to these risk factors [10, 25].

### Data synthesis and statistical analysis

We calculated the truncated age-standardized incidence rates (ASIRs) and age-standardized mortality rates (ASMRs) for four age groups (0‒14, 15‒49, 50‒74 and ≥ 75 years) using the WHO world standard population as the reference. The mortality-to-incidence ratio (MIR), a population-level indicator of cancer detection, management, and survival outcomes [26–28], was obtained by calculating the ratio of the ASMR to the ASIR. We used a Joinpoint regression model [29] to determine the temporal trends in the incidence and mortality of bladder, kidney, prostate, and testicular cancers during 1990–2021. The average annual percent changes (AAPCs) with 95% confidence intervals (CIs) for ASIRs and ASMRs of genitourinary cancers from 1990 to 2021 in the global, East Asia and Pacific, China, Japan, the Republic of Korea, the US, and the UK were determined using Joinpoint (version 4.9.1.0). When the *P-*values of the corresponding AAPC were < 0.05, the trends were considered to be increasing or decreasing; otherwise, the trends were considered stable. All analyses were performed with R software (version 4.3.3) for data management and visualization.

## Results

### Genitourinary cancer burden

**Fig. 1:**
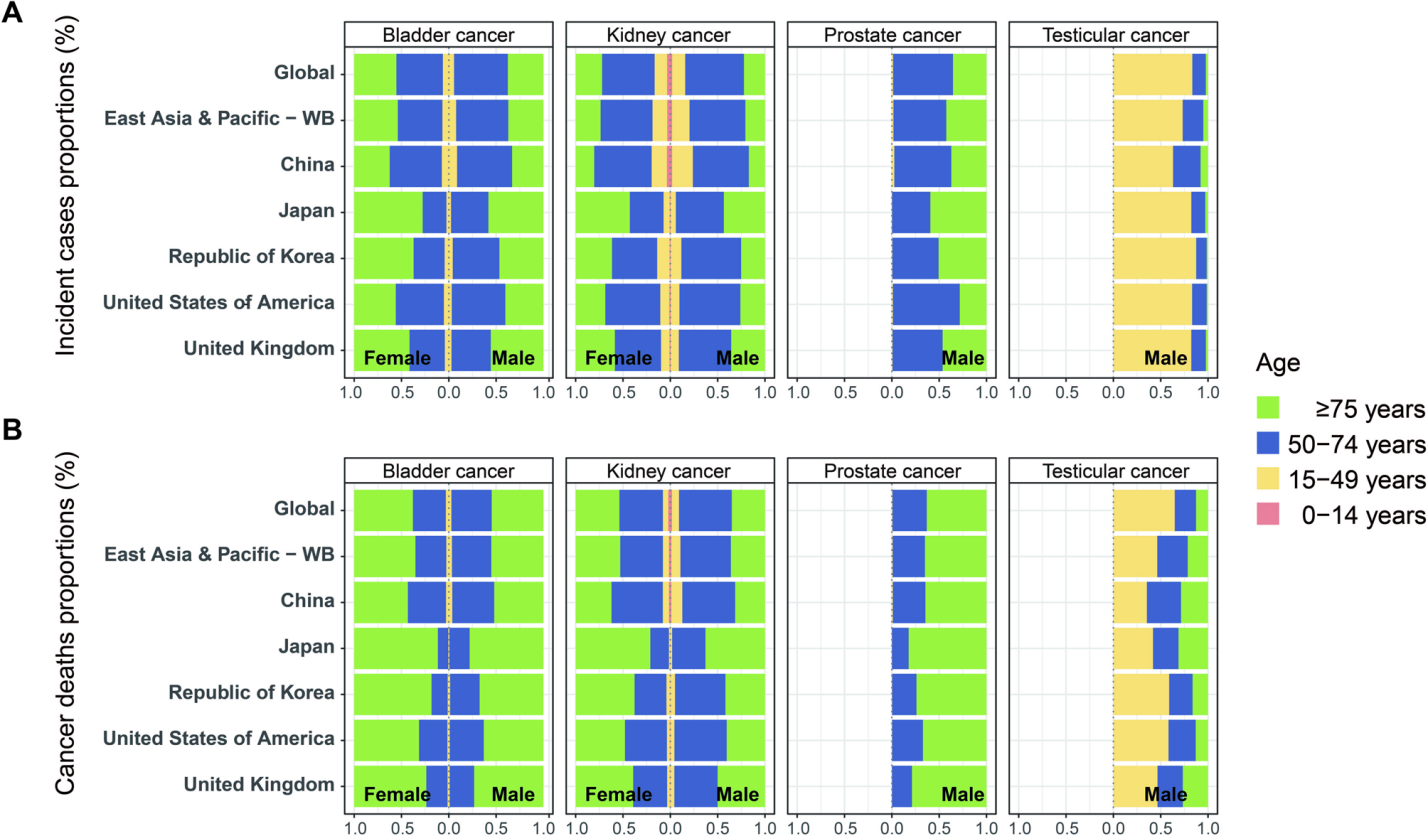
Age-specific proportions of incident and death cases of genitourinary cancers. (**A**) Proportions of incident cases by age group (0–14 years, 15–49 years, 50–74 years and ≥ 75 years) for bladder, kidney, prostate, and testicular cancers across global, East Asia and Pacific, China, Japan, the Republic of Korea, the US and the UK in GBD 2021. **(B)** Proportions of death cases by age group (0–14 years, 15–49 years, 50–74 years and ≥ 75 years) for bladder, kidney, prostate, and testicular cancers across global, East Asia and Pacific, China, Japan, the Republic of Korea, the US and the UK in GBD 2021

Table S1 shows the absolute number of incident cases of genitourinary cancers for four age groups in the global region, East Asia and Pacific region, China, Japan, the Republic of Korea, the US, and the UK. In 2021, China reported approximately 266,887 cases of genitourinary cancers, indicating a significant global and regional burden. Bladder, kidney, prostate, and testicular cancers in Chinese males represented 20.4%, 18.4%, 6.7%, and 7.3% of the global burden, respectively, and 65.4%, 63.1%, 38.5%, and 46.8% of the East Asia and Pacific region’s burden, respectively. Bladder and kidney cancer cases in Chinese females constituted 16.7% and 14.2% of the global cases, respectively, and 58.8% and 58.3% of East Asia and Pacific cases, respectively. Except for prostate and testicular cancers in males and bladder and kidney cancers in females, where case numbers in China were lower than those in the US, the overall number of genitourinary cancer cases in China exceeded those in other countries (Table S1). The distribution patterns across age groups in China for these cancers differed from those in Japan, the Republic of Korea, the US, and the UK (Table S1 and Fig. 1A). In China, both bladder and kidney cancers showed the greatest case numbers in the 50–74 age group, accounting for more than 50% of total cases across all age groups for both sexes. Prostate cancer in males also peaked in the same age range (50–74 years: 60.2%). Testicular cancer, on the other hand, had the highest incidence in the 15–49 age group (63.1%). The proportions of elderly patients (aged ≥ 75 years) with bladder, kidney, and prostate cancers in China were slightly lower than in other countries (all *P-*values < 0.05), whereas the proportion of elderly patients (aged ≥ 75 years) with testicular cancer was higher in China than in other countries (all *P-*values < 0.05, Table S1 and Fig. 1A).

Table S2 presents age-stratified mortality data for genitourinary cancers across four age groups, including the global, the East Asia and Pacific, and selected countries (China, Japan, the Republic of Korea, the US, and the UK). In 2021, there were 108,589 deaths from bladder, kidney, prostate, and testicular cancers in China. Mortality among Chinese males constituted 21.5% (bladder cancer), 16.6% (kidney cancer), 8.6% (prostate cancer), and 10.9% (testicular cancer) of the global burden and 67.0%, 58.4%, 42.4%, and 55.0% of the East Asia and Pacific region’s burden, respectively. Bladder and kidney cancer deaths in females represented 17.0% and 13.2% of the global burden and 56.6% and 52.3% of the East Asia and Pacific burden, respectively. Except for prostate cancer, for which deaths in China were lower than those in the US, deaths from other genitourinary cancers in China were higher for both sexes (Table S2). The distribution of genitourinary cancer deaths in China (Table S2 and Fig. 1B) exhibited distinct age-specific patterns: bladder cancer deaths in both sexes (52.0% male; 56.7% female) and prostate cancer deaths in males (64.5%) peaked in the ≥ 75 years age group, whereas kidney cancer deaths in both sexes (55.8% male; 54.3% female) and testicular cancer deaths in males (36.1%) were most frequent in the 50–74 years age group. Notably, compared with other countries and regions included in our analysis, China had higher proportions of deaths before age 75 from bladder and kidney cancers in both sexes and prostate cancer in males (all *P* values < 0.05, Table S2, Fig. 1B).

### Genitourinary cancer incidence and mortality rates

**Fig. 2:**
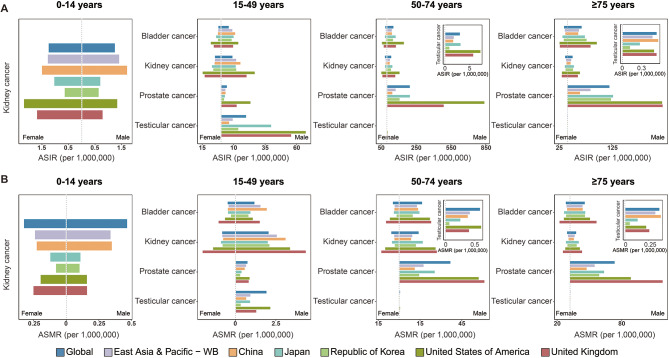
The ASIRs and ASMRs of genitourinary cancers by country and age groups. (**A**) The ASIRs of bladder, kidney, prostate, and testicular cancers for four age groups (0–14 years, 15–49 years, 50–74 years and ≥ 75 years) across global, East Asia and Pacific, China, Japan, the Republic of Korea, the US and the UK in GBD 2021. (**B**) The ASMRs of bladder, kidney, prostate, and testicular cancers for four age groups (0–14 years, 15–49 years, 50–74 years and ≥ 75 years) across global, East Asia and Pacific, China, Japan, the Republic of Korea, the US and the UK in GBD 2021. ASIRs, age-standardized incidence rates; ASMRs, age-standardized mortality rates

For all age groups, except for the ASIR of male bladder cancer in China (90.53), which was slightly higher than thatin East Asia and Pacific (89.25), China’s ASIRs for genitourinary cancers in both sexes were generally lower than those in the global, East Asia and Pacific, Japan, the Republic of Korea, the US, and the UK (Tables 1 and 2). However, in specific age groups, China had the highest ASIRs for male kidney cancer in the 0–14 years age group (China 1.70 vs. global 1.24, East Asia and Pacific 1.41, Japan 0.70, the Republic of Korea 0.63, the US 1.33, and the UK 0.78) and for male testicular cancer in the ≥ 75 years age group (China 0.56 vs. global 0.54, East Asia and Pacific 0.47, Japan 0.27, the Republic of Korea 0.12, the US 0.50, and the UK 0.54) compared with the studied regions and countries (Table 1; Fig. 2A). Additionally, in the 15–49 years age group, China’s ASIRs for male bladder cancer (11.22), kidney cancer (15.37), and prostate cancer (3.61) exceeded those of Japan (bladder cancer: 9.95, kidney cancer: 11.62, prostate cancer: 3.04) and the Republic of Korea (bladder cancer: 8.38, kidney cancer: 12.06, prostate cancer: 2.39) (Table 1; Fig. 2A). For females, China’s ASIR for kidney cancer (1.50) in the 0–14 years age group was higher than that in Japan (1.04) and the Republic of Korea (0.64) (Table 2; Fig. 2A).

**Table 1:** The ASIRs and ASMRs of male bladder, kidney, prostate, and testicular cancers across ages and locations in 2021 (per 1,000,000)

	**ASIRs**					**ASMRs**				
	All ages	0–14 years*	15–49 years	50–74 years	≥ 75 years	All ages	0–14 years*	15–49 years	50–74 years	≥ 75 years
Bladder cancer										
Global	109.19	0	6.07	55.80	39.74	46.66	0	1.18	16.48	22.79
	(100.37–119.05)	(0.00–0.00)	(5.36–6.90)	(51.27–61.14)	(35.71–43.24)	(42.17–51.62)	(0.00–0.00)	(1.03–1.35)	(14.94–18.68)	(20.40–25.15)
East Asia and Pacific-WB	89.25	0	8.93	41.99	32.19	39.79	0	1.55	12.78	19.32
	(74.44–108.96)	(0.00–0.00)	(7.14–11.32)	(33.69–53.24)	(27.39–37.91)	(33.24–47.94)	(0.00–0.00)	(1.23–1.97)	(10.11–16.30)	(16.21–23.10)
China	90.53	0	11.22	43.17	30.18	43.19	0	1.94	13.64	19.64
	(68.87–119.46)	(0.00–0.00)	(8.44–14.98)	(31.31–58.81)	(23.24–38.43)	(33.14–55.70)	(0.00–0.00)	(1.46–2.61)	(9.94–18.63)	(15.21–24.78)
Japan	123.42	0	9.95	74.93	48.90	37.71	0	0.93	14.58	23.56
	(113.19–131.20)	(0.00–0.00)	(9.11–10.96)	(69.18–80.68)	(42.27–53.24)	(33.71–39.95)	(0.00–0.00)	(0.90–0.96)	(13.84–15.24)	(20.10–25.36)
Republic of Korea	124.60	0	8.38	51.9	53.69	42.22	0	0.81	9.75	23.09
	(88.78–160.01)	(0.00–0.00)	(6.01–11.37)	(39.26–64.90)	(35.92–71.96)	(28.65–54.63)	(0.00–0.00)	(0.60–1.08)	(7.41–12.25)	(15.15–30.65)
US	240.43	0	13.68	145.87	82.78	57.98	0	1.08	22.13	30.99
	(224.18–251.59)	(0.00–0.00)	(13.12–14.33)	(140.03–151.59)	(72.83–88.37)	(52.26–61.40)	(0.00–0.00)	(1.03–1.12)	(21.21–23.02)	(26.75–33.27)
UK	153.95	0	9.56	77.22	64.99	71.06	0	1.51	22.99	40.98
	(143.91–160.73)	(0.00–0.00)	(9.15–9.97)	(74.07–80.23)	(58.21–68.78)	(64.80–74.49)	(0.00–0.00)	(1.45–1.56)	(22.11–23.83)	(36.43–43.33)
**Kidney cancer**										
Global	62.65	1.24	9.04	36.87	14.18	27.88	0.47	2.05	13.98	9.45
	(58.77–66.38)	(0.94–1.54)	(8.37–9.83)	(34.86–39.11)	(12.71–15.08)	(26.07–29.52)	(0.33–0.60)	(1.90–2.23)	(13.22–14.80)	(8.47–10.04)
East Asia and Pacific-WB	48.63	1.41	12.02	25.39	10.16	21.04	0.34	2.57	9.37	7.24
	(41.89–56.59)	(1.09–1.77)	(10.06–14.44)	(21.28–30.25)	(8.86–11.39)	(18.36–24.15)	(0.27–0.42)	(2.15–3.10)	(7.94–11.12)	(6.31–8.11)
China	47.95	1.70	15.37	23.93	8.47	19.18	0.35	3.11	8.56	5.93
	(37.76–60.14)	(1.20–2.29)	(11.92–19.37)	(18.11–31.10)	(6.74–10.23)	(15.31–23.92)	(0.24–0.47)	(2.41–3.93)	(6.48–11.12)	(4.80–7.16)
Japan	69.18	0.70	11.62	46.97	17.85	30.38	0.11	2.00	16.98	13.47
	(64.50–72.49)	(0.60–0.80)	(10.69–12.58)	(43.74–49.82)	(15.64–19.27)	(28.01–31.84)	(0.10–0.12)	(1.95–2.06)	(16.17–17.59)	(11.69–14.41)
Republic of Korea	61.71	0.63	12.06	35.04	15.29	26.61	0.10	2.10	12.07	10.36
	(51.97–71.92)	(0.36–0.96)	(9.12–15.10)	(28.67–41.68)	(12.39–18.36)	(22.38–30.91)	(0.06–0.16)	(1.62–2.62)	(9.88–14.35)	(8.50–12.38)
US	165.06	1.33	26.73	108.49	34.97	44.94	0.16	3.38	26.17	15.26
	(155.51–171.21)	(1.20–1.46)	(25.50–28.05)	(104.02–112.26)	(30.79–37.00)	(41.53–46.82)	(0.14–0.17)	(3.24–3.54)	(25.10–27.03)	(13.31–16.18)
UK	117.69	0.78	20.25	71.64	29.08	50.67	0.16	4.36	27.10	18.65
	(112.09–122.24	(0.71–0.87)	(19.38–21.26)	(68.74–74.29)	(26.13–30.77)	(47.53–52.71)	(0.14–0.17)	(4.20–4.54)	(26.15–28.02)	(16.73–19.71)
**Prostate cancer**										
Global	340.53	0	4.65	196.99	118.57	126.29	0	0.74	36.83	69.10
	(312.71–360.01)	(0.00–0.00)	(4.06–5.07)	(181.52–207.78)	(106.84–126.50)	(111.58–135.45)	(0.00–0.00)	(0.59–0.84)	(31.66–39.84)	(60.78–74.29)
East Asia and Pacific-WB	156.92	0	3.47	74.97	64.82	69.29	0	0.66	17.53	38.12
	(128.08–181.26)	(0.00–0.00)	(2.47–4.32)	(59.63–88.81)	(53.32–73.91)	(55.31–81.44)	(0.00–0.00)	(0.45–0.80)	(13.28–21.35)	(30.29–44.27)
China	93.39	0	3.61	46.37	35.15	48.92	0	0.57	11.19	25.77
	(66.81–125.72)	(0.00–0.00)	(2.45–4.89)	(33.19–64.48)	(24.38–46.36)	(35.83–65.26)	(0.00–0.00)	(0.39–0.78)	(8.20–15.72)	(18.57–34.24)
Japan	297.49	0	3.04	197.48	128.89	76.92	0	0.38	25.55	52.75
	(261.01–329.05)	(0.00–0.00)	(2.34–3.88)	(166.04–234.98)	(107.05–143.97)	(67.71–81.83)	(0.00–0.00)	(0.36–0.39)	(23.93–26.68)	(44.79–56.83)
Republic of Korea	257.80	0	2.39	108.96	123.22	76.75	0	0.30	14.38	45.19
	(154.89–341.96)	(0.00–0.00)	(1.46–3.70)	(69.50–154.46)	(71.00–164.89)	(44.34–99.75)	(0.00–0.00)	(0.18–0.42)	(9.41–19.71)	(25.74–59.13)
US	1080.37	0	23.37	838.37	267.95	164.76	0	0.88	57.20	94.19
	(1014.81–1130.37)	(0.00–0.00)	(21.66–25.26)	(798.15–877.81)	(236.84–285.70)	(146.63–175.11)	(0.00–0.00)	(0.83–0.92)	(54.72–59.44)	(80.83–101.34)
UK	761.04	0	12.37	487.91	269.41	228.29	0	0.82	61.28	143.49
	(711.15–796.91)	(0.00–0.00)	(11.36–13.48)	(460.41–515.51)	(243.36–285.05)	(206.44–239.29)	(0.00–0.00)	(0.79–0.85)	(58.92–63.56)	(127.19–151.23)
**Testicular cancer**										
Global	22.45	0	19.86	3.06	0.54	2.87	0	1.92	0.60	0.36
	(21.59–23.47)	(0.00–0.00)	(19.06–20.79)	(2.89–3.26)	(0.48–0.59)	(2.70–3.04)	(0.00–0.00)	(1.81–2.04)	(0.56–0.64)	(0.33–0.40)
East Asia and Pacific-WB	10.94	0	9.22	1.82	0.47	1.68	0	0.92	0.43	0.32
	(9.66–12.35)	(0.00–0.00)	(8.25–10.24)	(1.51–2.24)	(0.39–0.57)	(1.43–1.95)	(0.00–0.00)	(0.78–1.08)	(0.35–0.52)	(0.27–0.38)
China	8.20	0	6.37	1.69	0.56	1.52	0	0.67	0.39	0.38
	(6.38–10.48)	(0.00–0.00)	(5.04–8.02)	(1.25–2.30)	(0.43–0.71)	(1.18–1.91)	(0.00–0.00)	(0.51–0.84)	(0.29–0.52)	(0.30–0.47)
Japan	42.49	0	39.77	3.20	0.27	1.23	0	0.90	0.26	0.13
	(38.27–46.90)	(0.00–0.00)	(35.39–44.48)	(2.79–3.59)	(0.24–0.30)	(1.18–1.27)	(0.00–0.00)	(0.87–0.94)	(0.25–0.27)	(0.12–0.14)
Republic of Korea	13.89	0	13.67	0.91	0.12	0.43	0	0.32	0.07	0.05
	(10.08–18.75)	(0.00–0.00)	(9.78–18.34)	(0.62–1.32)	(0.07–0.20)	(0.32–0.60)	(0.00–0.00)	(0.24–0.43)	(0.05–0.10)	(0.03–0.08)
US	71.47	0	67.24	7.28	0.50	2.89	0	2.16	0.62	0.22
	(68.10–75.05)	(0.00–0.00)	(63.79–70.86)	(6.92–7.63)	(0.45–0.53)	(2.78–2.99)	(0.00–0.00)	(2.07–2.24)	(0.60–0.64)	(0.20–0.24)
UK	58.20	0	55.65	5.80	0.54	1.88	0	1.29	0.41	0.26
	(55.51–60.66)	(0.00–0.00)	(53.07–58.22)	(5.48–6.13)	(0.48–0.57)	(1.81–1.94)	(0.00–0.00)	(1.25–1.33)	(0.39–0.43)	(0.23–0.27)

*Incidence and mortality data provide in GBD 2021 for bladder cancer, prostate cancer, and testicular cancer in aged 0–14 are 0

ASIRs, age-standardized incidence rates; ASMRs, age-standardized mortality rate

**Table 2:** The ASIRs and ASMRs of female bladder and kidney cancers across ages and locations in 2021 (per 1,000,000)

	ASIRs					ASMRs				
	All ages	0–14 years*	15–49 years	50–74 years	≥ 75 years	All ages	0–14 years*	15–49 years	50–74 years	≥ 75 years
Bladder cancer										
Global	26.44	0	2.06	13.46	10.00	12.23	0	0.48	4.46	6.50
	(23.40–28.90)	(0.00–0.00)	(1.88–2.29)	(12.52–14.55)	(8.21–11.14)	(10.58–13.44)	(0.00–0.00)	(0.43–0.54)	(4.14–4.83)	(5.31–7.26)
East Asia and Pacific-WB	19.87	0	2.21	9.33	7.76	9.73	0	0.43	3.21	5.39
	(16.36–23.47)	(0.00–0.00)	(1.81–2.70)	(7.74–11.22)	(6.05–9.11)	(7.91–11.41)	(0.00–0.00)	(0.35–0.52)	(2.68–3.81)	(4.21–6.31)
China	18.87	0	2.48	9.74	6.32	9.19	0	0.47	3.41	4.54
	(14.37–23.91)	(0.00–0.00)	(1.77–3.37)	(7.39–12.52)	(4.74–7.95)	(7.03–11.50)	(0.00–0.00)	(0.34–0.64)	(2.60–4.34)	(3.42–5.66)
Japan	30.74	0	3.91	16.45	14.44	11.40	0	0.46	3.78	9.27
	(25.33–34.11)	(0.00–0.00)	(3.58–4.22)	(14.47–18.00)	(10.15–17.15)	(8.69–12.92)	(0.00–0.00)	(0.45–0.48)	(3.33–4.05)	(6.36–11.01)
Republic of Korea	22.03	0	2.39	8.17	10.74	8.82	0	0.28	1.82	5.98
	(16.27–26.81)	(0.00–0.00)	(1.84–3.06)	(6.00–10.40)	(7.36–13.60)	(6.11–10.88)	(0.00–0.00)	(0.22–0.36)	(1.34–2.30)	(3.99–7.62)
US	70.64	0	8.09	42.40	22.74	16.67	0	0.65	6.69	9.53
	(63.38–74.50)	(0.00–0.00)	(7.72–8.50)	(39.65–44.23)	(18.03–25.13)	(14.15–17.99)	(0.00–0.00)	(0.62–0.68)	(6.23–6.98)	(7.45–10.60)
UK	53.89	0	5.76	27.47	21.82	26.58	0	1.05	9.43	16.24
	(48.44–56.81)	(0.00–0.00)	(5.53–6.01)	(26.07–28.56)	(17.95–23.81)	(23.09–28.53)	(0.00–0.00)	(1.01–1.09)	(8.94–9.82)	(13.16–17.84)
**Kidney cancer**										
Global	29.96	1.25	4.77	16.76	6.90	11.90	0.32	0.82	5.62	4.62
	(27.60–31.98)	(1.02–1.55)	(4.42–5.18)	(15.64–17.76)	(5.68–7.56)	(10.58–12.75)	(0.25–0.42)	(0.76–0.89)	(5.24–5.94)	(3.80–5.07)
East Asia and Pacific-WB	20.43	1.28	4.79	10.33	4.20	8.07	0.24	0.82	3.55	3.15
	(17.03–24.31)	(0.98–1.60)	(3.91–5.90)	(8.58–12.46)	(3.21–4.95)	(6.69–9.54)	(0.19–0.29)	(0.68–0.99)	(2.95–4.26)	(2.39–3.70)
China	19.47	1.50	5.10	10.10	3.10	6.87	0.23	0.76	3.39	2.25
	(14.39–25.21)	(1.02–2.07)	(3.60–6.92)	(7.50–13.32)	(2.30–4.02)	(5.12–8.85)	(0.15–0.32)	(0.54–1.05)	(2.51–4.46)	(1.66–2.93)
Japan	28.63	1.04	7.17	16.07	7.88	10.89	0.12	0.90	5.14	6.34
	(24.79–31.70)	(0.80–1.32)	(6.06–8.50)	(13.95–18.06)	(5.52–9.36)	(8.88–12.09)	(0.11–0.13)	(0.88–0.92)	(4.58–5.47)	(4.40–7.51)
Republic of Korea	21.98	0.64	6.13	10.31	5.68	8.27	0.08	0.80	3.14	3.97
	(17.32–26.38)	(0.39–1.08)	(4.52–8.21)	(7.88–12.76)	(3.76–7.61)	(6.13–10.20)	(0.05–0.12)	(0.60–1.03)	(2.43–3.82)	(2.66–5.23)
US	76.93	2.18	14.58	47.84	15.97	18.48	0.20	1.39	10.06	7.35
	(71.37–80.42)	(2.00–2.35)	(13.79–15.44)	(44.90–49.85)	(12.98–17.51)	(16.31–19.55)	(0.18–0.21)	(1.33–1.45)	(9.46–10.44)	(5.84–8.11)
UK	66.88	1.68	12.97	38.40	16.53	25.62	0.25	2.03	12.81	10.99
	(62.23–70.36)	(1.50–1.89)	(12.22–13.82)	(36.40–40.44)	(13.92–18.09)	(23.18–27.17)	(0.23–0.28)	(1.97–2.10)	(12.23–13.38)	(9.05–12.05)

*Incidence and mortality data provide in GBD 2021 for bladder cancer in aged 0–14 are 0

ASIRs, age-standardized incidence rates; ASMRs, age-standardized mortality rates

China’s ASMRs for the four genitourinary cancers were also generally lower than the global, East Asia and Pacific averages, as well as those in Japan, the Republic of Korea, the US, and the UK. However, several exceptions were observed (Tables 1 and 2). For example, China’s ASMR for male bladder cancer (43.19) was higher than those in East Asia and Pacific region (39.79), Japan (37.71), and the Republic of Korea (42.22). Additionally, China’s ASMR for male testicular cancer (1.52) was higher than that of Japan (1.23) and the Republic of Korea (0.43) (Table 1). China’s ASMR for female bladder cancer (9.19) was slightly higher than that in the Republic of Korea (8.82) (Table 2). Among the specific age groups, China had the highest ASMRs for male bladder cancer in the 15–49 years age group (China 1.94 vs. global 1.18, East Asia and Pacific 1.55, Japan 0.93, the Republic of Korea 0.81, US 1.08, and UK 1.51) and for male testicular cancer in the ≥ 75 years age group (China 0.38 vs. global 0.36, East Asia and Pacific 0.32, Japan 0.13, the Republic of Korea 0.05, the US 0.22, and the UK 0.26) compared with other regions and countries. Additionally, China’s ASMR for male kidney cancer in the 0–14 years age group was 0.35, which was lower than the global value of 0.47 but higher than those of Japan (0.11), the Republic of Korea (0.10), the US (0.16), and the UK (0.16) (Table 1; Fig. 2B). For female kidney cancer in the same age group (0–14 years), China’s ASMR was 0.23, which was lower than that of the global (0.32) and the UK (0.25) but higher than those of Japan (0.12), the Republic of Korea (0.08), and the US (0.20) (Table 2; Fig. 2B). Both male and female ASMRs for kidney cancer in China’s 0–14 years age group were comparable to those in East Asia and the Pacific’s level (Tables 1 and 2; Fig. 2B). For males aged 15–49 years, China’s ASMRs for kidney cancer (3.11) and prostate cancer (0.57) were higher than those in Japan (kidney cancer: 2.00, prostate cancer: 0.38) and the Republic of Korea (kidney cancer: 2.10, prostate cancer: 0.30) (Table 1; Fig. 2B).

**Fig. 3:**
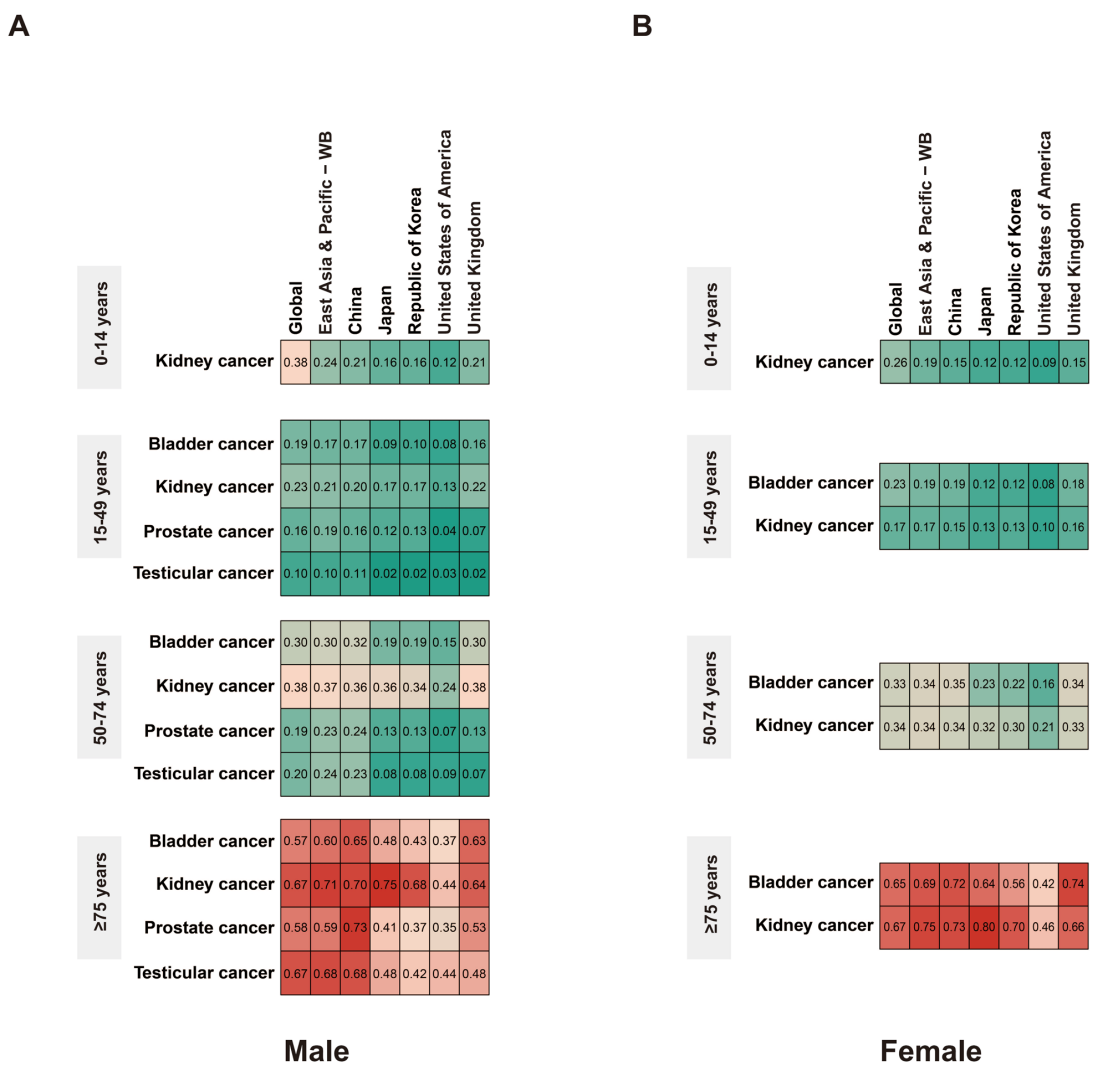
The MIRs of genitourinary cancers by countries and age groups. (**A**) The male MIRs of bladder, kidney, prostate, and testicular cancers for four age groups (0–14 years, 15–49 years, 50–74 years and ≥ 75 years) across global, East Asia and Pacific, China, Japan, the Republic of Korea, the US and the UK in GBD 2021. **(B)** The female MIRs of bladder, kidney, prostate, and testicular cancers for four age groups (0–14 years, 15–49 years, 50–74 years and ≥ 75 years) across global, East Asia and Pacific, China, Japan, the Republic of Korea, the US and the UK in GBD 2021. MIRs, mortality-to-incidence ratios

China also had a relatively high incidence and mortality burden of bladder and kidney cancers in males. The male-to-female ratios of ASIRs and ASMRs for bladder cancer in the 15–49 and ≥ 75 years age groups and for kidney cancer across the 0–14, 15–49, and ≥ 75 years age groups were higher in China than in other countries and regions (Table S3). The MIRs for all genitourinary cancers in both sexes followed a similar pattern: they increased with age and peaked in the ≥ 75 years age group (Fig. 3A-B). In China, the MIRs for genitourinary cancers were generally similar to the global and East Asia and Pacific levels, except for lower MIRs for kidney cancer in children (0–14 years) and higher MIRs for bladder and prostate cancers in the elderly (≥ 75 years). Except for kidney cancer in both sexes in the ≥ 75 years age group and in male aged 50–74 years, where MIRs were lower than or equal to those in Japan, China’s MIRs for genitourinary cancers across all age groups were higher than those in Japan and the Republic of Korea. Compared with the US and the UK, China’s MIRs for bladder and kidney cancers in both sexes were similar to those in the UK but significantly higher than those in the US. Additionally, China had much higher MIRs for male prostate and testicular cancers across all age groups than the US and the UK (Fig. 3A-B).

### Trends in genitourinary cancer incidence and mortality rates

**Fig. 4:**
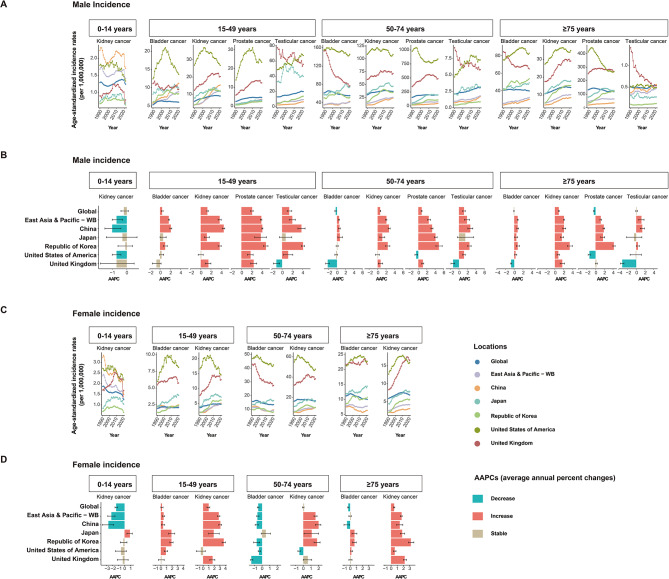
Trends in annual ASIRs of genitourinary cancers from 1990 to 2021. **(A)** Temporal trends in ASIRs of male bladder, kidney, prostate, and testicular cancers for four age groups (0–14 years, 15–49 years, 50–74 years and ≥ 75 years) across global, East Asia and Pacific, China, Japan, the Republic of Korea, the US and the UK from 1990 to 2021. **(B)** AAPCs in the ASIRs of male bladder, kidney, prostate, and testicular cancers across age groups (0–14 years, 15–49 years, 50–74 years and ≥ 75 years) and geographic locations (global, East Asia and Pacific, China, Japan, the Republic of Korea, the US and the UK). **(C)** Temporal trends in ASIRs of female bladder, kidney, prostate, and testicular cancers for four age groups (0–14 years, 15–49 years, 50–74 years and ≥ 75 years) across global, East Asia and Pacific, China, Japan, the Republic of Korea, the US and the UK from 1990 to 2021. **(D)** AAPCs in the ASIRs of female bladder, kidney, prostate, and testicular cancers across age groups (0–14 years, 15–49 years, 50–74 years and ≥ 75 years) and geographic locations (global, East Asia and Pacific, China, Japan, the Republic of Korea, the US and the UK). AAPCs, average annual percent changes; ASIRs, age-standardized incidence rates

From 1990 to 2021, the ASIRs of genitourinary cancers exhibited varying trends across countries and regions (Fig. 4A-D and Table S4). For ASIRs of kidney cancer in the 0–14 years age group, an increasing trend was noted exclusively among Japanese females (AAPC of 0.87%), whereas globally and in East Asia and Pacific, as well as other countries, it either declined or stabilized from 1990 to 2021. In China, the historically high ASIRs of kidney cancer for both sexes aged 0–14 years decreased significantly (with AAPCs of -0.98% for males and − 2.73% for females). Despite this decline, China’s ASIRs for male kidney cancer in the 0–14 years age group remained above those of all other regions and countries. Moreover, China’s ASIR for female kidney cancer in the same age group (0–14 years) fell below those of Japan and the UK with rapid declines, yet was still higher than those of the US and the Republic of Korea (Fig. 4C-D and Table S4). In contrast, for the 15–49, 50–74, and ≥ 75 years age groups, kidney cancer ASIRs increased in most countries and regions, except for the US, which showed stable or declining trends in the 15–49 and 50–74 years age groups. Notably, in China, for males aged 15–49 years, the AAPC for kidney cancer was significantly higher (4.48% vs. global 1.34%, East Asia and Pacific 3.69%, Japan 1.19%, the Republic of Korea 3.72%, US 0%, and UK 1.48%), and thus the ASIR surpassed other regions and countries, ranking just behind two Western nations (Fig. 4A-B and Table S4). For bladder cancer, the ASIRs in the two Western countries had remained high over the past three decades, with a downward or stable trend observed across the age groups (15–49, 50–74, and ≥ 75 years) in the UK, in contrast with a slight upward trend in both sexes aged ≥ 75 years and females aged 15–49 years and ≥ 75 years in the US. Although the ASIRs of bladder cancer in East Asia and Pacific and three Asian countries were relatively lower from 1990 to 2021, the increasing trends in male ASIRs were pronounced, with the AAPCs in the three Asian countries much higher than those in Western nations across the same age groups (15–49, 50–74, ≥ 75 years). Notably, the incidence rate among Chinese males aged 15–49 showed an accelerated increase (AAPC of 2.14%) compared with the global of 0.41%, East Asia and Pacific of 1.70%, Japan of 0.58%, the Republic of Korea of 1.04%, the US of 0.33%, and the UK of -0.66%, leading China’s ASIR to rank second only to the US (Fig. 4A-D and Table S4). For prostate cancer, with the exception of the US in the 50–74 years age group, as well as in those aged ≥ 75 years in the global, the US and the UK, all other studied regions and countries showed increasing trends of ASIRs across all age groups (15–49, 50–74, and ≥ 75 years) from 1990 to 2021. Notably, East Asia and Pacific regions showed a more pronounced increase in prostate cancer incidence, with the Republic of Korea leading in AAPCs, followed by China and Japan across all age groups (15–49, 50–74, and ≥ 75 years) (Fig. 4A-B and Table S4). For testicular cancer, the two Western nations maintained higher incidence rates than other countries and regions. In contrast to the UK and Japan, which experienced declining or stable ASIRs during the study period, China and the Republic of Korea showed increasing trends with higher AAPCs. Despite starting from the lower rates, China’s ASIRs of testicular cancer have risen to be the highest among countries in the ≥ 75 years age group, with an AAPC of 1.43% (Fig. 4A-B and Table S4).

**Fig. 5:**
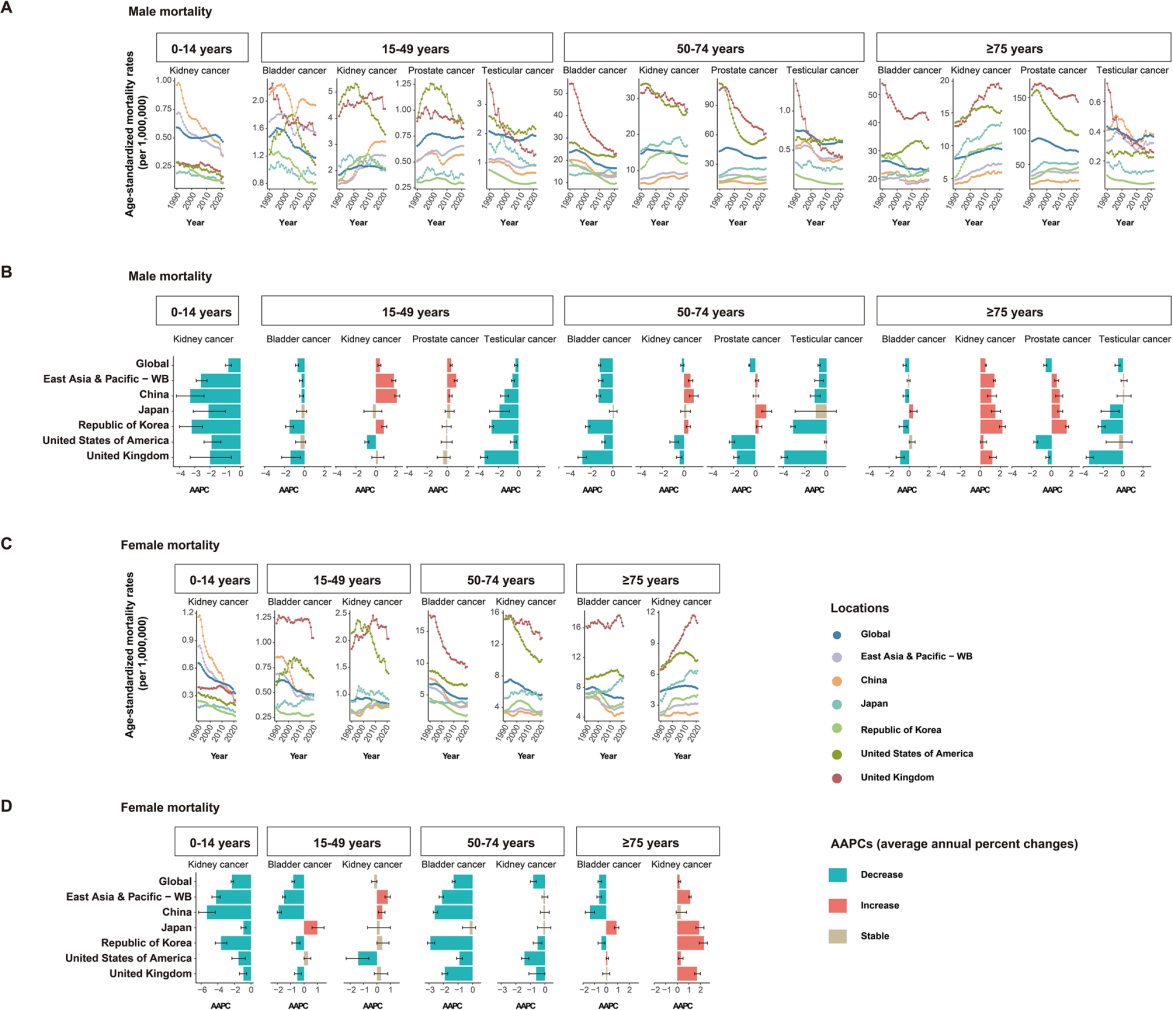
Trends in annual ASMRs of genitourinary cancers from 1990 to 2021. **(A)** Temporal trends in ASMRs of male bladder, kidney, prostate, and testicular cancers for four age groups (0–14 years, 15–49 years, 50–74 years and ≥ 75 years) across global, East Asia and Pacific, China, Japan, the Republic of Korea, the US and the UK from 1990 to 2021. **(B)** AAPCs in the ASMRs of male bladder, kidney, prostate, and testicular cancers across age groups (0–14 years, 15–49 years, 50–74 years and ≥ 75 years) and geographic locations (global, East Asia and Pacific, China, Japan, the Republic of Korea, the US and the UK). **(C)** Temporal trends in ASMRs of female bladder, kidney, prostate, and testicular cancers for four age groups (0–14 years, 15–49 years, 50–74 years and ≥ 75 years) across global, East Asia and Pacific, China, Japan, the Republic of Korea, the US and the UK from 1990 to 2021. **(D)** AAPCs in the ASMRs of female bladder, kidney, prostate, and testicular cancers across age groups (0–14 years, 15–49 years, 50–74 years and ≥ 75 years) and geographic locations (global, East Asia and Pacific, China, Japan, the Republic of Korea, the US and the UK). AAPCs, average annual percent changes; ASMRs, age-standardized mortality rates

Notable differences in ASMRs trends were observed between Asian and Western nations from 1990 to 2021 (Fig. 5A-D and Table S4). For kidney cancer, all studied regions and countries exhibited significant decreases in ASMRs for both sexes aged 0–14 years, with China experiencing the largest decrease (an AAPC of -3.33% for males and − 5.30% for females). Except for Japan, Asian countries reported an increasing trend in ASMRs of kidney cancer for males aged 15–49 and 50–74 years, which contrasted with the decreasing and stable trends in Western countries. Notably, China’s rapidly increasing rates (AAPC: 2.13%) of kidney cancer mortality in males aged 15–49 caused the trend line to steeply increase, surpassing both Japan and the Republic of Korea and approaching the levels of the US. In the ≥ 75 years age group, all regions and countries, with the exception of China in terms of female kidney cancer, experienced significantly increasing trends in ASMRs for both sexes (Fig. 5A-D and Table S4). For bladder cancer, increasing trends in ASMRs were observed for females aged 15–49 years (with an AAPC of 1.02%) and both sexes aged ≥ 75 years (with AAPCs of 0.44% for males and 0.92% for females) in Japan, whereas other regions and countries showed declining or stable trends. Despite a downward trend, China’s bladder cancer mortality in the 15–49 years male group remained higher than that in other countries (Fig. 5A-D and Table S4). For prostate cancer, mortality in Western countries exceeded those in the global and Asian countries, yet the Western countries displayed general decline or stable trends, whereas East Asia and pacific region showed a marked upturn in mortality across all age groups. Among Asians countries studied, China’s 15–49 years age group showed increasing trends, leading to higher mortality rates than those of Japan and the Republic of Korea, which showed stable trends similar to those of Western countries (Fig. 5A-B and Table S4). Testicular tumor mortality trended downward in all countries and regions across all age groups; however, in China’s ≥ 75 years age group, the fluctuating mortality rates masked a higher mortality rate than those in Japan, the Republic of Korea, and Western countries (Fig. 5A-B and Table S4).

**Fig. 6:**
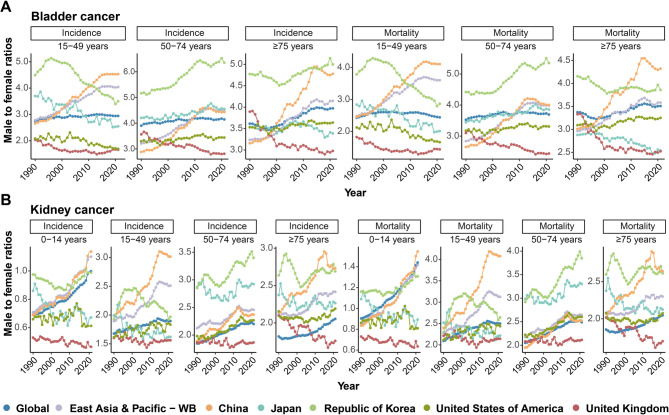
Trends of male-to-female ratios of ASIRs and ASMRs in bladder and kidney cancers. **(A)** Trends of male-to-female ratios in bladder cancer incidence and mortality rates across age groups (15–49 years, 50–74 years and ≥ 75 years) and geographic locations (global, East Asia and Pacific, China, Japan, the Republic of Korea, the US and the UK) from 1990 to 2021. **(B)** Trends of male-to-female ratios in kidney cancer incidence and mortality rates across age groups (0–14 years, 15–49 years, 50–74 years and ≥ 75 years) and geographic locations (global, East Asia and Pacific, China, Japan, the Republic of Korea, the US and the UK) from 1990 to 2021

**Fig. 7:**
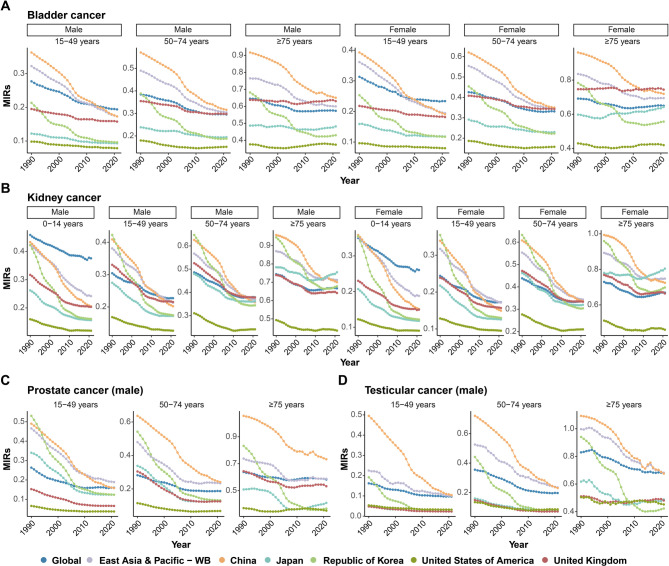
Trends of MIRs in genitourinary cancers across sexes, locations, and ages from 1990 to 2021. **(A)** Trends of MIRs in bladder cancer across sexes (male and female), age groups (15–49 years, 50–74 years and ≥ 75 years), and geographic locations (global, East Asia and Pacific, China, Japan, the Republic of Korea, the US and the UK) from 1990 to 2021. **(B)** Trends of MIRs in kidney cancer across sexes (male and female), age groups (0–14 years, 15–49 years, 50–74 years and ≥ 75 years), and geographic locations (global, East Asia and Pacific, China, Japan, the Republic of Korea, the US and the UK) from 1990 to 2021. **(C)** Trends of MIRs in prostate cancer across age groups (15–49 years, 50–74 years and ≥ 75 years), and geographic locations (global, East Asia and Pacific, China, Japan, the Republic of Korea, the US and the UK) from 1990 to 2021. (**D**) Trends of MIRs in testicular cancer across age groups (15–49 years, 50–74 years and ≥ 75 years), and geographic locations (global, East Asia and Pacific, China, Japan, the Republic of Korea, the US and the UK) from 1990 to 2021. MIRs, mortality to incidence ratios

China experienced a significant widening of sex differences in both bladder and kidney cancers in terms of incidence and mortality across all age groups. The trends in the male-to-female ratios for incidence (ASIRs) and mortality (ASMRs) increased and were higher than those in other countries and regions, particularly in the 15–49 and ≥ 75 years age groups for bladder cancer and in the 0–14, 15–49, and ≥ 75 years age groups for kidney cancer (Fig. 6A-B). Regarding the trends in the MIRs (Fig. 7A-D), despite the generally decreasing trends observed in China and other countries and regions, China still presented higher MIRs for genitourinary cancers, especially in the 50–74 and ≥ 75 years age groups for male bladder cancer and for prostate cancer and in the 15–49, 50–74, and ≥ 75 years age groups for testicular cancer.

### Decomposition analysis

**Fig. 8:**
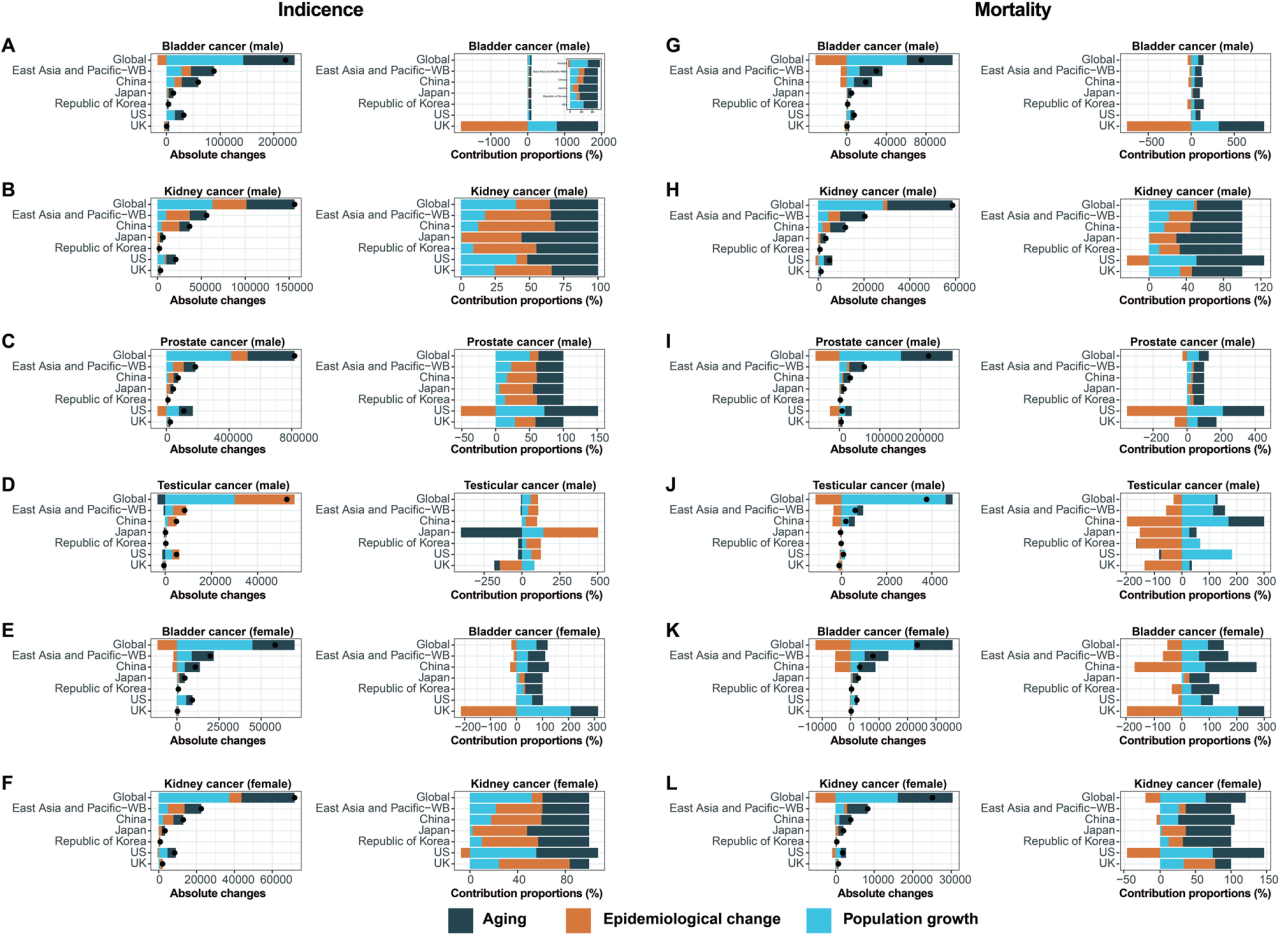
Decomposition analysis on changes in genitourinary cancers incidence and mortality across sexes and locations from 1990 to 2021. Absolute changes and contribution proportions of **(A)** male kidney cancer incidence, (**B**) male bladder cancer incidence, (**C**) male prostate cancer incidence, (**D**) male testicular cancer incidence, (**E**) female kidney cancer incidence, and (**F**) female bladder cancer incidence attributed to the three factors (aging, population growth, and epidemiological change). Absolute changes and contribution proportions of (**G**) male kidney cancer mortality, (**H**) male bladder cancer mortality, (**I**) male prostate cancer mortality, (**J**) male testicular cancer mortality, (**K**) female kidney cancer mortality, and (**L**) female bladder cancer mortality attributed to the three factors (aging, population growth, and epidemiological change). The black dot in A-L represents the combined effect of all three factors, including aging, population growth, and epidemiological change

The decomposition analysis revealed that the increase in incident cases of genitourinary cancers in most regions and countries from 1990 to 2021 was attributable to aging, population growth, and epidemiological changes, which collectively contributed to a growing burden of these cancers over time (Fig. 8A-F and Table S5).

Globally, population growth was the primary driver of the increase in the incidence of genitourinary cancers (bladder cancer: males 64.41%, females 76.89%; kidney cancer: males 40.12%, females 51.93%; prostate cancer: 50.61%; and testicular cancer: 57.06%, Fig. 8A-F and Table S5). In East Asia and Pacific, aging was the main positive driver for the increase in the incidence of bladder cancer in both males (48.47%) and females (66.03%), as well as for prostate cancer (40.06%) and female kidney cancer (39.23%), whereas epidemiological changes primarily affect the increase in incidence of male kidney cancer (48.18%) and testicular cancer (67.27%, Fig. 8A-F and Table S5). In three Asian countries, aging made the greatest contribution to the increase in the incidence of bladder cancer in both males and females, with contributions of 51.80% in men and 81.58% in women in China, 69.03% in men and 68.69% in women in Japan, and 64.90% in men and 66.78% in women in the Republic of Korea (Fig. 8A and E, and Table S5). In China (males 55.94% and females 42.18%) and the Republic of Korea (males 46.08% and females 47.33%), the increase in the incidence of male and female kidney cancer was also largely due to epidemiological changes, whereas in Japan, aging was the main factor (males 55.81% and females 52.04%, Fig. 8B and F, and Table S5). The increase in male prostate and testicular cancer incidence was driven predominantly by epidemiological changes in China (44.44% and 76.45%), Japan (49.67% and 361.74%), and the Republic of Korea (47.42% and 95.75%, Fig. 8C-D and Table S5). In the US, the increase in the incidence of male bladder (51.14%), kidney (51.67%), and prostate (79.33%) cancers was driven mainly by aging, with testicular cancer being strongly influenced by epidemiological changes (62.84%) and female bladder (60.22%) and kidney (55.72%) cancers caused by population growth (Fig. 8A-F and Table S5). The UK observed an increase in the incidence of kidney cancer, which was driven primarily by contributions from epidemiological changes in both males (41.40%) and females (59.29%). Aging exerted a substantial positive influence on the increase in the incidence of male bladder (1115.72%) and prostate (41.21%) cancers, while population growth was the main driver of female bladder (208.87%) cancers (Fig. 8A-F and Table S5).

According to the decomposition analysis, population growth was the major positive driver for the increases in mortality from bladder cancer in males (80.64%) and females (95.14%), prostate cancer (69.06%) and testicular cancer (122.34%) in males, and kidney cancer (64.26%) in females globally (Fig. 8G-L and Table S6). Population growth also primarily impacted the increase in testicular cancer mortality in East Asia and Pacific (114.39%), China (170.14%), and the US (183.11%), as well as for female bladder cancer in the US (69.67%) and the UK (206.07%), and for female kidney cancer in the US (74.30%, Fig. 8J-L and Table S6). Aging was the main positive driver of increased bladder cancer mortality in males in East Asia and Pacific (76.75%), China (94.78%), Japan (80.26%), the Republic of Korea (109.74%), the US (56.21%), and the UK (526.60%, Fig. 8G and Table S6). Male kidney cancer (global 48.54%, East Asia and Pacific 53.09%, China 55.55%, Japan 70.58%, Republic of Korea 66.94%, the US 72.57%, and the UK 53.82%, Fig. 8H and Table S6) and prostate cancer (global 57.85%, East Asia and Pacific 60.10%, China 64.95%, Japan 69.00%, Republic of Korea 61.49%, the US 242.29%, and the UK 110.48%, Fig. 8I and Table S6) in all studied regions and countries, as well as female bladder and kidney cancer in East Asia and Pacific (106.24% and 63.87%) and three Asian countries (bladder cancer: China 186.66%, Japan 72.23%, and the Republic of Korea 101.96%; kidney cancer: China 79.60%, Japan 63.75%, and the Republic of Korea 67.82%) were also predominantly driven by aging (Fig. 8K-L and Table S6). Although epidemiological changes contributed positively to the increased mortality of male kidney and prostate cancers, as well as female kidney cancers in Asian regions and countries, they generally contributed negatively to the overall mortality burden of all genitourinary cancers in Western countries (Fig. 8G-L and Table S6). This negative impact was particularly pronounced for testicular cancer mortality (Fig. 8J and Table S6), where the negative contributions of epidemiological changes surpassed the positive increases attributed to population growth and aging, resulting in the observed decreases in the numbers of testicular cancer-related deaths in Japan (-38), the Republic of Korea (-10), and the UK (-101).

### Risk factor trends

**Fig. 9:**
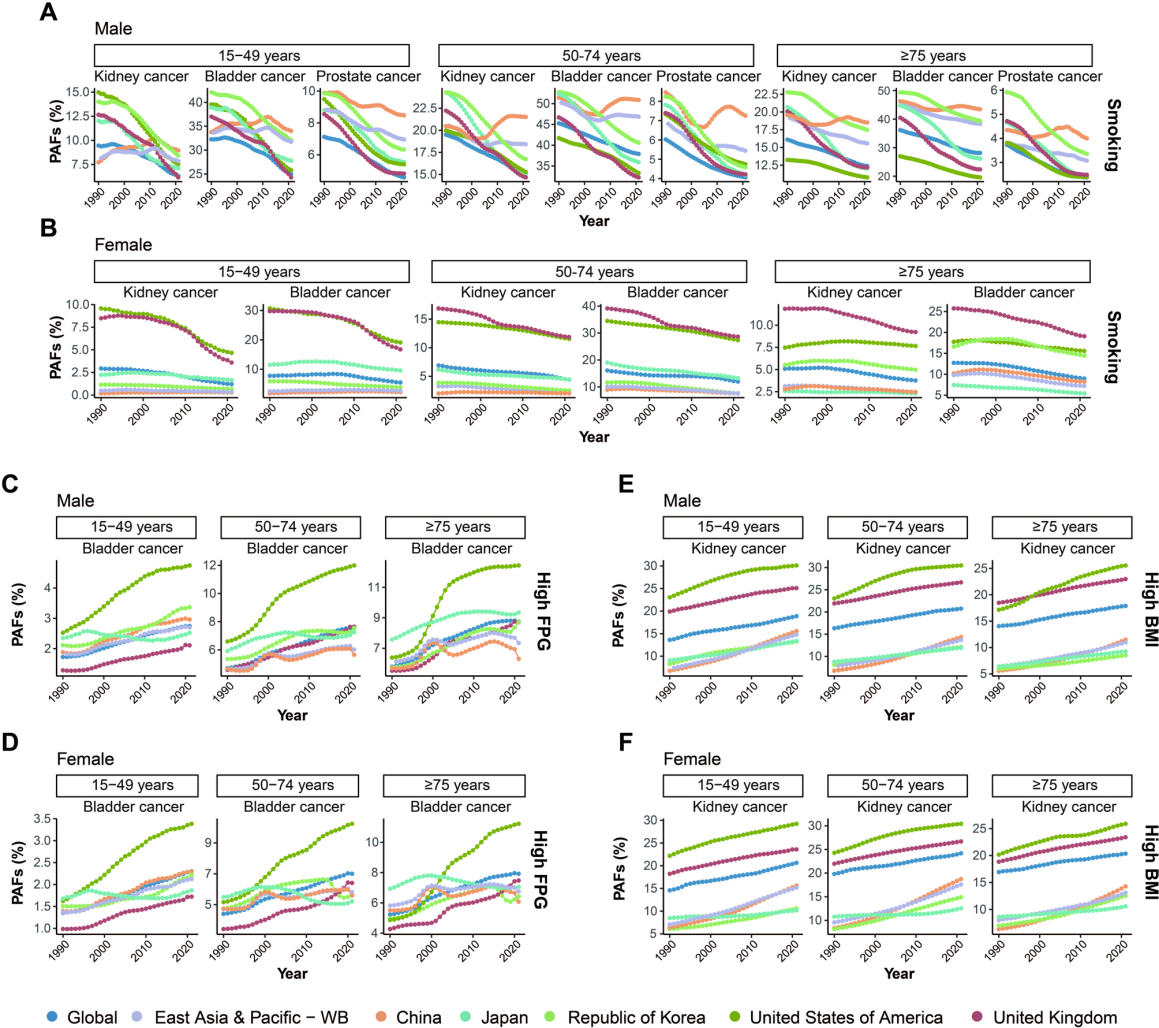
PAFs of genitourinary cancers mortality due to risk factors across sexes, locations, and ages from 1990 to 2021. **(A)** PAFs of smoking-related mortality for male kidney, bladder, and prostate cancers across age groups (15–49 years, 50–74 years and ≥ 75 years) and geographic locations (global, East Asia and Pacific, China, Japan, the Republic of Korea, the US and the UK) from 1990 to 2021. **(B)** PAFs of smoking-related mortality for female kidney, bladder, and prostate cancers across age groups (15–49 years, 50–74 years and ≥ 75 years) and geographic locations (global, East Asia and Pacific, China, Japan, the Republic of Korea, the US and the UK) from 1990 to 2021. **(C)** PAFs of high FPG-related mortality for male bladder cancer across age groups (15–49 years, 50–74 years and ≥ 75 years) and geographic locations (global, East Asia and Pacific, China, Japan, the Republic of Korea, the US and the UK) from 1990 to 2021. **(D)** PAFs of high FPG-related mortality for female bladder cancer across age groups (15–49 years, 50–74 years and ≥ 75 years) and geographic locations (global, East Asia and Pacific, China, Japan, the Republic of Korea, the US and the UK) from 1990 to 2021. (**E)** PAFs of high BMI-related mortality for male kidney cancer across age groups (15–49 years, 50–74 years and ≥ 75 years) and geographic locations (global, East Asia and Pacific, China, Japan, the Republic of Korea, the US and the UK) from 1990 to 2021. (**F**) PAFs of high BMI-related mortality for female kidney cancer across age groups (15–49 years, 50–74 years and ≥ 75 years) and geographic locations (global, East Asia and Pacific, China, Japan, the Republic of Korea, the US and the UK) from 1990 to 2021. BMI, body mass index; FPG, fasting plasma glucose; PAFs, population-attributable fractions

The GBD did not provide risk factors for testicular cancer mortality. Therefore, in our study, we analyzed the impacts of smoking, a common modifiable risk factor, on kidney, bladder, and prostate cancers (Fig. 9A-B and Figure S1A-C). Additionally, we investigated the effects of a high FPG level on bladder cancer (Fig. 9C-D and Figure S2) and a high BMI on kidney cancer (Fig. 9E-F and Figure S3). Among these cancers, bladder cancer consistently recorded the highest PAFs for smoking-related mortality across all age groups and for both sexes. Notably, Chinese males showed an increase in PAFs for kidney, bladder, and prostate cancers, which contrasts with the general downward trend observed in other countries. Moreover, China continues to exhibit significantly higher PAFs for smoking-related mortality in male genitourinary cancers (Fig. 9A-B**)**. For high FPG, an overall increasing trend in PAFs was observed for bladder cancer mortality in the 15–49 years age group in both sexes in China (Fig. 9C-D). Furthermore, a high BMI was identified as another major risk factor for kidney cancer mortality in China, with increasing trends observed across all age groups and for both sexes. China’s PAFs of high BMI-related kidney cancer mortality were second only to those globally, the US, and the UK (Fig. 9E-F).

The attributable numbers of kidney, bladder, and prostate cancer-related deaths due to smoking are displayed in Figure S1A-C. Smoking-related mortality was more common in the 50–74 and ≥ 75 years age groups. Other than the 15–49 and 50–74 years age groups, increasing trends in attributable numbers of male kidney, bladder, and prostate cancer mortality related to smoking were observed in the ≥ 75 years age group globally, in the East Asia and Pacific, and in the three Asian countries, with the highest increase in attributable numbers for bladder cancer. Compared with other countries, China exhibited a distinct upward trend in smoking-attributable mortality for these cancers in the 50–74 years age group. Although China displayed similar trends in mortality attributable to a high FPG and a high BMI as other countries (Figure S2 and Figure S3), the attributable numbers for bladder cancer mortality related to high FPG remained consistently greater across all age groups and for both sexes.

## Discussion

The burden and trends of genitourinary cancers globally, in the East Asia and Pacific region, and in China, Japan, Republic of Korea, the US, and the UK across the 0–14, 15–49, 50–74, and ≥ 75 years age groups were systematically analyzed in this study utilizing the latest high-quality updated GBD 2021 study [10]. Compared with the global, the East Asia-Pacific region, as well as two neighboring Asian countries and two developed Western countries, China has a considerable absolute incidence and mortality for kidney, bladder, prostate, and testicular cancers, with distinct age distribution patterns. Although China’s overall incidence and mortality rates for genitourinary cancers are relatively low, specific age groups present higher rates. In terms of temporal trends, the incidence of genitourinary cancers in China shows a marked upward trend, whereas mortality rates, except for bladder cancer, which shows a declining trend, demonstrate varying degrees of increase for other genitourinary cancers. These trends underscore the need for targeted public health interventions due to the impacts of aging, population growth, and epidemiological changes in the genitourinary cancer burden. We found that mortality from smoking-related genitourinary cancers was still higher in Chinese males. Additionally, mortality related to a high BMI for kidney cancer among Chinese males and females over the age of 15 years and mortality related to a high FPG level for bladder cancer among both sexes in China within the 15–49 years age group showed increasing trends. These findings highlight the urgent need for strategic health policies in China to combat the increasing burden of genitourinary cancers, emphasizing risk factor modification, early detection, and enhanced health care access, especially for the most vulnerable age groups, consistent with the country’s unique epidemiological characteristics.

High-income North America, Western Europe, and East Asia have the highest rates of prostate cancer and account for the majority of new cases of bladder and kidney cancers. East Asia, in particular, has shown the most significant increase in the ASIR for bladder and kidney cancers, as well as the fastest increase in the ASMR for kidney cancer [4]. Additionally, a more than twofold increase in the incidence of testicular cancer in East Asia has been documented [2]. These trends underscore the importance of comparative analyses between specific high-burden countries, as they can reveal disparities in cancer risk factors, early detection capabilities, and the effectiveness of health policies. In recent decades, China’s cancer patterns have shifted significantly, mirroring those of developed countries [30, 31]. In particular, the prevalence of genitourinary cancers has increased [3, 4], a pattern previously more common in Western countries. By focusing on age-specific populations, our results revealed higher proportions of incidence and mortality cases for bladder, kidney, and prostate cancers among patients under the age of 75 years and for testicular cancer in those over 50 years in China. Conversely, in the global and East Asia and Pacific regions, as well as countries such as Japan, the Republic of Korea, the US, and the UK, higher proportions of incidence and mortality are observed for bladder, kidney, and prostate cancers in those aged above 75 years, and for testicular cancer in those under 50 years. The varying pace and degree of aging across countries may be a contributing factor to the differences in the age distribution described above [32]. China is still relatively “young” [33], in comparison, countries such as Japan, the Republic of Korea, the US, and the UK, appear to be aging much more severely. Japan is among the most severely aged countries globally, with a significantly higher proportion of people aged 65 years and older [34]. The Republic of Korea is also rapidly aging and is projected to enter a “superaged society” by 2025 [35, 36]. Moreover, the US, a developed nation, faces notable aging issues [37, 38]. This variation in age distribution, which has also been reported for other cancers in China [39], indicates the need for continued attention to age differences in the burden of genitourinary cancers and the effectiveness of intervention strategies. The accelerating aging of China’s population is anticipated to exert a substantial effect on the demographic structure, leading to a dual burden of genitourinary cancers across younger and older age groups in the near future. This shift is set to create a distinctive pattern of cancer incidence, with significant implications for public health strategies and the necessity for targeted interventions.

Currently, regional and country disparities exist in the incidence and mortality rates of genitourinary cancers, with Western countries generally exhibiting higher rates than Asia [4, 40]. Although Asian regions typically have lower incidence rates of genitourinary cancers, a growing trend exists that contrasts with the stabilizing or declining rates observed in Western countries [40–42]. This divergence underscores the importance of developing region-specific cancer control strategies that consider local epidemiological profiles. Relying solely on data from Western populations may not provide an adequate understanding of cancer trends, particularly in regard to the different patterns observed in specific age groups. Our study reveals that the incidence rates of bladder, kidney, and prostate cancers are notably higher among Chinese men under 50 years of age. This result is further emphasized by the fact that Chinese males aged 0–14 years have unusually high kidney cancer rates, which are declining, and men aged 15–49 years are experiencing a sharp increase in bladder, kidney, and prostate cancer rates, nearing Western levels. Additionally, testicular cancer incidence rates among men over 75 years of age in China have also increased, surpassing those reported in other studied countries. Mortality rates also show concerning trends; for men under 50 years and over 75 years, kidney and prostate cancer mortality rates are increasing in China, which contrasts with Western trends. Although the exact underlying reasons have not been fully elucidated, these variations further suggest a greater burden of genitourinary cancers among both younger working-age men and elderly men in China. This finding highlights the urgent need for further investigation to address the unique challenges faced by different age groups in China.

The main drivers of overall changes in the genitourinary cancer burden due to aging, population growth, and changes in rates vary by region and country. The decomposition analysis revealed that aging is the primary driver of the increasing incidence of bladder cancer in most of the regions and countries studied. This result aligns with epidemiological findings that identify advanced age as the predominant risk factor for bladder cancer [43]. The risk is further exacerbated by exposure to carcinogens such as tobacco smoke and less prevalent but potent substances such as benzene and aromatic amines, compounded by an age-related decline in DNA repair efficiency [44]. All three Asian countries in our study are encountering similar demographic transitions and are likely to experience similar rapid increases in the burdens of kidney, prostate, and testicular cancers, where increasing incidence is a major factor. These divergent regional trends underscore the need for localized cancer strategies over generalizing from Western data. Furthermore, the decomposition analysis indicates that population growth and aging are the leading drivers of increased mortality from genitourinary cancers. As life expectancy continues to rise—projected to increase from 73.6 years in 2022 to 78.2 years by 2050 [45, 46]—the burden of genitourinary cancers is expected to become even more pronounced. The impending increase in aging populations in Asian countries calls for proactive policy-making and strategic planning to safeguard the health and well-being of specific age groups.

Smoking, high FPG level, and obesity (high BMI) each contribute significantly to China’s genitourinary cancer burden, with distinct demographic patterns. Smoking remains the leading modifiable risk factor, particularly for bladder cancer (the highest PAF), although its attributable mortality in Chinese men, while declining, has persisted at higher levels than in other countries despite tobacco control efforts since 2005 [47]. Concurrently, a high FPG level drives bladder cancer mortality, especially among younger adults (15–49 years), with PAFs nearing Western levels, which is likely exacerbated by urbanization-related metabolic shifts, and potentially synergistic effects with smoking and obesity [48, 49]. Most strikingly, a high BMI now surpasses smoking as a risk factor for kidney cancer mortality in both sexes, with rapidly rising PAFs exceeding those reported in Japan and the Republic of Korea [50]. These trends underscore the urgent need for integrated, sex- and age-specific strategies targeting smoking cessation, metabolic health, and obesity prevention in China’s evolving cancer landscape.

Differences in cancer incidence rates and mortality rates between men and women have been widely reported [51, 52]. Our analysis highlighted notable sex disparities in the incidence and mortality rates of bladder and kidney cancers, with men typically having higher rates than women across all age groups and countries. This pattern is particularly pronounced in bladder cancer, aligning with existing global research [1, 53]. Nonetheless, a notable exception occurs in kidney cancer among females aged 0–14 years in countries such as Japan, the US, and the UK, where slightly higher incidence and mortality rates are observed than in males aged 0–14 years. Currently, systematic analyses of the variability in sex ratios across specific countries and age groups for bladder and kidney cancers remain lacking. The stable male predominance of kidney and bladder cancers across different age groups is influenced by a multitude of factors. In addition to sex chromosomes and hormones, distinct biological mechanisms and epigenetic differences underlie the fundamental sex disparities in these cancers [51, 54, 55]. Risk factors, which are examined in our study, also contribute to these disparities [56, 57]. These factors are not independent but interact in complex ways to influence sex differences in cancer susceptibility and outcomes. The modifiable nature of these risk factors underscores their importance in cancer prevention and management. Our research indicates that, compared with other countries, China has a more significant sex disparity in the burden of bladder and kidney cancers. While the need to focus on male cancer management is justified, recognizing that the link between smoking and mortality from bladder and kidney cancer in women, although relatively low, may be underestimated due to typically lower smoking rates among females is crucial [6]. This complexity is further compounded by previous reports of delays in both presentation and diagnostic workup for female patients with bladder and kidney cancer [58, 59]. Moreover, the influence of a high BMI on kidney cancer mortality seems to be more pronounced in females than in males. Therefore, emphasizing the importance of smoking cessation and weight control for both sexes is essential to mitigate the burden of bladder and kidney cancer.

The MIR is a valuable population-based indicator that assesses disparities in cancer diagnosis, treatment, and survival [26–28]. In our study, the findings that MIRs for genitourinary cancers are relatively high in China compared with other countries suggest a greater burden of these cancers within the Chinese population. Notably, higher MIRs in older age groups (≥ 75 years) indicate that the impact of genitourinary cancers may be more pronounced on this demographic group. This result could be attributed to specific factors, including biological differences [60], such as functional disability, polypharmacy, malnutrition, age-related pharmacological differences in the metabolism of anticancer treatments, systemic metabolic changes, and a weaker immune response to cancer, as well as social and health care access disparities in older adults [61] that can affect the timeliness and effectiveness of cancer treatment in this age group. The high MIRs for genitourinary cancers in China may also reflect the rapid aging of the population, which, as highlighted in our study, is expected to further increase the incidence and mortality of age-related cancers such as bladder, kidney, prostate, and testicular cancers in the coming decade. This finding underscores the need for the timely development and implementation of optimal health policies to address the increasing burden of these cancers. Furthermore, the observed MIRs also reflect disparities in early detection across countries. For example, the widespread prostate-specific antigen (PSA) testing in the US since the 1990s has significantly increased early-stage detection but also revealed substantial overdiagnosis [62], prompting revised USPSTF recommendations [63]. While the ERSPC trial reported a 21% overdiagnosis rate [64], the Swedish Göteborg cohort demonstrated a concurrent 29% reduction in mortality [65], highlighting the need for balanced implementation. Japan’s population-wide PSA screening had been associated with declining mortality despite its increasing incidence [66], whereas China’s current low screening coverage is reflected in its high metastatic diagnosis rate (30.5% at presentation [67]). These disparities underscore how health care infrastructure and policy influence screening outcomes. China’s urban‒rural disparities in health care access and uneven insurance coverage complicate standardized PSA implementation. The Chinese Anti-Cancer Association now advocates shared decision-making [68], emphasizing risk-adapted screening protocols, active surveillance for low-risk cases, and targeted awareness campaigns in high-burden regions. Our findings reflect current screening practices but caution that increasing PSA adoption may necessitate future PAF adjustments, that registry-based monitoring of overdiagnosis trends is critical, and that MRI-guided biopsy protocols [69] could mitigate the risk of overdiagnosis. Enhanced health care policies and heightened public awareness about disease prevention are also crucial for reducing the burden of genitourinary cancers [6, 70]. The broader application of advanced abdominal imaging technologies in developed countries has also led to the increased early detection of kidney cancer [5, 70]. Moreover, a positive correlation exists between the incidence of bladder cancer and the level of development among countries [6], which may be attributed to better disease awareness and improved access to health care systems, facilitating the earlier identification of bladder cancer in more affluent nations. The incidence of testicular cancer has steadily increased worldwide over the past 30 years, particularly in young men. In 2020, the highest incidence rates were noted in Western countries and Japan [71]. Our study revealed a consistent increase in the incidence and mortality rates of testicular cancer across all age groups in China, especially in older age groups (≥ 75 years) in 2021. A similar increase in mortality in older individuals has also been recently reported in the US [72]. The fact that risk factors for testicular cancer are not well understood [2, 73] underscores the need for further large-scale cohort studies and the development of targeted strategies for prevention and early intervention. This could include an in-depth examination of lifestyle habits, genetic predispositions, and environmental exposures that may contribute to the development of testicular cancer. Although the predicted incidence rates of some genitourinary cancers increase, with the progress of early diagnosis and treatment strategies, the mortality may decline [74–78].

The strength of this study is the use of the most up-to-date comprehensive epidemiological data for four genitourinary cancers, including kidney, bladder, prostate, and testicular cancers across the global, East Asia and Pacific region, the US, the UK, China, the Republic of Korea, and Japan from 1990 to 2021. In addition, this study reveals long-term temporal changes in genitourinary cancer patterns and burdens by concentrating on four specific age groups and sexes. Moreover, our decomposition analysis provides a clearer understanding of the multifaceted factors influencing the burden of genitourinary cancers, particularly in the context of an aging global population. Finally, we examined the changes in the impact of behavioral risk factors (smoking) and two metabolic risk factors (a high BMI and high FPG levels) on the genitourinary cancer burden between 1990 and 2021. Our study, while providing valuable insights, has certain limitations. First, it lacks detailed information on the histological subtypes of genitourinary cancers, which is crucial for a comprehensive analysis. Second, the GBD 2021 data, which we relied upon, include only a limited set of risk factors for genitourinary cancers and may not account for the entire range of etiological factors involved. Third, the COVID-19 pandemic has introduced considerable uncertainty in the estimation of mortality rates across all diseases, especially in regions hardest hit by the pandemic. This finding underscores the need for continuous surveillance and research to fully understand the long-term impacts of the pandemic on cancer mortality estimates.

## Conclusions

In summary, China exhibits unique age-specific patterns, with notably higher incidence and mortality rates in certain age groups than the global average, the East Asia and Pacific regions (including Japan and the Republic of Korea), and Western developed countries such as the US and the UK. The incidence of genitourinary cancers in China has shown a marked upward trend over time, influenced by population aging, epidemiological shifts, and associated risk factors. Our study reveals a notable increase in the attribution of smoking to mortality from genitourinary cancers among Chinese males, along with significant sex differences and higher MIRs in the burden of these cancers. These findings underscore the urgent need for targeted interventions, risk factor management, and the implementation of adaptable cancer control programs to address the growing burden of genitourinary cancers in China.

## Supplementary Information

Below is the link to the electronic supplementary material.

Supplementary Material 1

Supplementary Material 2

Supplementary Material 3

Supplementary Material 4

Supplementary Material 5

Supplementary Material 6

## Data Availability

No datasets were generated or analysed during the current study.

## Abbreviations

ASIR: Age-standardized incidence rate
ASMR: Age-standardized mortality rate
US: United States
UK: United Kingdom
AAPC: Average annual percentage change
FPG: Fasting plasma glucose
BMI: Body mass index
GBD: Global Burden of Disease
PAF: Population-attributable fractions
MIR: Mortality-to-Incidence Ratio
