# Supplementary material for: Incidence, mortality, and risk factors of bladder, kidney, prostate and testicular cancers in China and comparisons with the United States, the United Kingdom, Japan, and the Republic of Korea: an up-to-date overview based on the Global Burden of Disease 2021
Source: Exp Hematol Oncol. 2025 Aug 6;14:103. doi: 10.1186/s40164-025-00694-9 (PMC12329898; doi:10.1186/s40164-025-00694-9)
Supplement: Supplementary file 1 — Supplementary Material 1 [file 40164_2025_694_MOESM1_ESM.docx]

# Supplementary methods

**Decomposition Analysis**

Aging, population growth, and epidemiological changes are key population-level factors utilized in decomposition analysis to determine their individual contributions to shifts in incidence and mortality at the population level. Aging is generally estimated by alterations in the age structure, while population growth indicates an increase in the total population size. In the context of decomposition analysis for incidence, epidemiological change refers to variations in the incidence rate; for mortality, it refers to changes in the mortality rate.

In conducting a decomposition analysis, taking incidence as an example, the number of incident cases is derived from the following equation:

Incidence _ay, py, ey_ = $\sum_{i=1}^{4}$( a_i,y_ * p_y_* e_i,y_). ​

Here, a_i,y_ represents the proportion of the population in age category i out of the 4 age categories (0-14 years, 15-49 years, 50-74 years, and ≥75 years) in year y; p_y_ denotes the total population in year y; and e_i,y_ signifies the genitourinary cancer incidence rate for age category i in year y. The net contribution of each factor to the total change in incidence is ascertained by examining the effect of one factor while keeping the others constant. The proportion of the total change attributed to each factor is calculated using the formula:

Proportion= $\frac{Absolute value of change driven by the factor}{Absolute value of the overall change}$ *.*

The proportion is positive when the change driven by the factor is positive, and negative otherwise.

The net contribution of **aging** was calculated as:

($\frac{\mathrm{Incidence}a2021, p1990, e1990 + Incidencea2021, p2021, e2021}{3}$ + $\frac{\mathrm{Incidence}a2021, p1990, e2021 + Incidencea2021, p2021, e1990}{6}$) -

($\frac{\mathrm{Incidence}a1990, p2021, e2021 + Incidencea1990, p1990, e1990}{3}$ + $\frac{\mathrm{Incidence}a1990, p2021, e1990 + Incidencea1990, p1990, e2021}{6}$)

The net contribution of **population growth** was calculated as:

($\frac{\mathrm{Incidence}a1990, p2021, e1990 + Incidencea2021, p2021, e2021}{3}$ + $\frac{\mathrm{Incidence}a1990, p2021, e2021 + Incidencea2021, p2021, e1990}{6}$) -

($\frac{\mathrm{Incidence}a1990, p1990, e1990 + Incidencea2021, p1990, e2021}{3}$ + $\frac{\mathrm{Incidence}a1990, p1990, e2021 + Incidencea2021, p1990, e1990}{6}$)

The net contribution of **incidence rate** was calculated as:

($\frac{\mathrm{Incidence}a1990, p1990, e2021 + Incidencea2021, p2021, e2021}{3}$ + $\frac{\mathrm{Incidence}a1990, p2021, e2021 + Incidencea2021, p1990, e1990)}{6}$) -

($\frac{\mathrm{Incidence}a1990, p1990, e1990 + Incidencea2021, p2021, e1990}{3}$ + $\frac{\mathrm{Incidence}a1990, p2021 e1990 + Incidencea2021, p1990, e1990}{6}$)
