# Supplementary material for: Incidence, mortality, and risk factors of bladder, kidney, prostate and testicular cancers in China and comparisons with the United States, the United Kingdom, Japan, and the Republic of Korea: an up-to-date overview based on the Global Burden of Disease 2021
Source: Exp Hematol Oncol. 2025 Aug 6;14:103. doi: 10.1186/s40164-025-00694-9 (PMC12329898; doi:10.1186/s40164-025-00694-9)
Supplement: Supplementary file 2 — Supplementary Material 2 [file 40164_2025_694_MOESM2_ESM.docx]

# Supplementary tables

**Table S1.** The incident cases of four genitourinary cancers in 2021

**Table S2.** The death cases of four genitourinary cancers in 2021

**Table S3.** The male to female ratios of incidence and mortality in genitourinary cancers varies by age, regions and countries in 2021

**Table S4.** Trends in annual ASIRs and ASMRs by across ages and locations, 1990-2021

**Table S5.** Decomposition analysis on changes in genitourinary cancers incidence across sexes and locations from 1990 to 2021

**Table S6.** Decomposition analysis on changes in genitourinary cancers mortality across sexes and locations from 1990 to 2021

**Table S7.** Relative risks of smoking, high BMI, and high FPG for genitourinary cancers

# Table S1. The incident cases of four genitourinary cancers in 2021

|  | **Global,**  **n(%)** | **East Asia and Pacific-WB,**  **n(%)** | **China,**  **n(%)** | **Japan,**  **n(%)** | **Republic of Korea,**  **n(%)** | **US,**  **n(%)** | **UK,**  **n(%)** |
| --- | --- | --- | --- | --- | --- | --- | --- |
| **Male** |  |  |  |  |  |  |  |
| **Bladder cancer** |  |  |  |  |  |  |  |
| 0-14 years* | 0 (0) | 0 (0) | 0 (0) | 0 (0) | 0 (0) | 0 (0) | 0 (0) |
| 15-49 years | 23330.9 (5.6) | 10139.63 (7.8) | 7436.71 (8.7) | 493.99 (2.4) | 204.57 (4.2) | 2006.69 (3.1) | 275.7 (2.9) |
| 50-74 years | 237458.27 (56.8) | 71991.21 (55.1) | 49629.56 (58.2) | 8297.95 (39.6) | 2421.79 (49.3) | 36597.08 (56.7) | 4003.27 (41.6) |
| ≥75 years | 156916.78 (37.6) | 48421.01 (37.1) | 28252.47 (33.1) | 12185.15 (58.1) | 2285.67 (46.5) | 25973.58 (40.2) | 5353.37 (55.6) |
| Total | 417705.96 (100.0) | 130551.85 (100.0) | 85318.74 (100.0) | 20977.09 (100.0) | 4912.03 (100.0) | 64577.35 (100.0) | 9632.34 (100.0) |
| **Kidney cancer** |  |  |  |  |  |  |  |
| 0-14 years | 4936.95 (2.0) | 1322.59 (1.8) | 903.26 (1.9) | 21.11 (0.2) | 7.54 (0.3) | 154.88 (0.4) | 18.04 (0.3) |
| 15-49 years | 34764.33 (13.8) | 13650.27 (18.5) | 10186.51 (21.9) | 576.53 (5.6) | 294.42 (11.4) | 3920.82 (9.3) | 584.26 (8.7) |
| 50-74 years | 156907.11 (62.1) | 43533.00 (59.0) | 27511.29 (59.1) | 5202.16 (50.8) | 1634.88 (63.2) | 27218.76 (64.4) | 3713.74 (55.3) |
| ≥75 years | 55980.79 (22.2) | 15274.52 (20.7) | 7927.64 (17.0) | 4448.35 (43.4) | 650.72 (25.1) | 10973.32 (26.0) | 2395.13 (35.7) |
| Total | 252589.19 (100.0) | 73780.38 (100.0) | 46528.70 (100.0) | 10248.15 (100.0) | 2587.56 (100.0) | 42267.78 (100.0) | 6711.16 (100.0) |
| **Prostate cancer** |  |  |  |  |  |  |  |
| 0-14 years* | 0 (0) | 0 (0) | 0 (0) | 0 (0) | 0 (0) | 0 (0) | 0 (0) |
| 15-49 years | 17865.06 (1.3) | 3937.49 (1.7) | 2388.81 (2.7) | 150.68 (0.3) | 58.27 (0.6) | 3427.13 (1.2) | 356.98 (0.7) |
| 50-74 years | 838314.83 (63.3) | 128522.66 (55.9) | 53312.39 (60.2) | 21870.70 (40.4) | 5083.72 (48.9) | 210339.99 (70.6) | 25294.30 (52.9) |
| ≥75 years | 468203.01 (35.4) | 97494.82 (42.4) | 32899.85 (37.1) | 32115.31 (59.3) | 5245.36 (50.5) | 84068.95 (28.2) | 22192.52 (46.4) |
| Total | 1324382.90 (100.0) | 229954.97 (100.0) | 88601.06 (100.0) | 54136.69 (100.0) | 10387.35 (100.0) | 297836.07 (100.0) | 47843.80 (100.0) |
| **Testicular cancer** |  |  |  |  |  |  |  |
| 0-14 years* | 0 (0) | 0 (0) | 0 (0) | 0 (0) | 0 (0) | 0 (0) | 0 (0) |
| 15-49 years | 76363.74 (83.5) | 10468.83 (73.2) | 4223.68 (63.1) | 1973.75 (82.4) | 333.62 (87.5) | 9863.14 (83.3) | 1605.49 (82.3) |
| 50-74 years | 13026.27 (14.2) | 3114.70 (21.8) | 1946.64 (29.1) | 354.61 (14.8) | 42.51 (11.2) | 1826.48 (15.4) | 300.50 (15.4) |
| ≥75 years | 2117.37 (2.3) | 709.29 (5.0) | 525.41 (7.8) | 68.32 (2.9) | 5.12 (1.3) | 155.75 (1.3) | 44.08 (2.3) |
| Total | 91507.38 (100.0) | 14292.81 (100.0) | 6695.73 (100.0) | 2396.68 (100.0) | 381.25 (100.0) | 11845.37 (100.0) | 1950.07 (100.0) |
| **Female** |  |  |  |  |  |  |  |
| **Bladder cancer** |  |  |  |  |  |  |  |
| 0-14 years* | 0 (0) | 0 (0) | 0 (0) | 0 (0) | 0 (0) | 0 (0) | 0 (0) |
| 15-49 years | 7722.86 (6.3) | 2365.87 (6.8) | 1517.31 (7.4) | 186.72 (2.5) | 53.35 (4.5) | 1177.25 (5.2) | 169.84 (4.3) |
| 50-74 years | 60230.80 (49.1) | 16402.66 (47.1) | 11260.92 (55.0) | 1889.25 (25.2) | 390.44 (32.7) | 11502.97 (50.9) | 1485.61 (37.3) |
| ≥75 years | 54650.11 (44.6) | 16058.61 (46.1) | 7693.54 (37.6) | 5421.36 (72.3) | 749.22 (62.8) | 9915.45 (43.9) | 2329.47 (58.5) |
| Total | 122603.77 (100.0) | 34827.14 (100.0) | 20471.77 (100.0) | 7497.33 (100.0) | 1193.01 (100.0) | 22595.66 (100.0) | 3984.92 (100.0) |
| **Kidney cancer** |  |  |  |  |  |  |  |
| 0-14 years | 4639.01 (3.4) | 1086.71 (3.3) | 694.13 (3.6) | 29.74 (0.6) | 7.29 (0.7) | 241.90 (1.1) | 37.06 (0.9) |
| 15-49 years | 17867.10 (13.2) | 5128.92 (15.5) | 3124.58 (16.2) | 342.75 (6.6) | 136.60 (13.2) | 2122.42 (9.5) | 382.57 (9.0) |
| 50-74 years | 75020.57 (55.5) | 18166.11 (54.9) | 11677.52 (60.6) | 1845.64 (35.6) | 492.89 (47.7) | 12979.43 (58.2) | 2076.27 (48.7) |
| ≥75 years | 37712.85 (27.9) | 8696.18 (26.3) | 3774.52 (19.6) | 2959.90 (57.2) | 396.35 (38.4) | 6962.52 (31.2) | 1764.89 (41.4) |
| Total | 135239.53 (100.0) | 33077.93 (100.0) | 19270.75 (100.0) | 5178.03 (100.0) | 1033.13 (100.0) | 22306.27 (100.0) | 4260.80 (100.0) |

*Incident cases data provide in GBD 2021 for bladder cancer, prostate cancer, and testicular cancer in aged 0-14 are 0.

# Table S2. The death cases of four genitourinary cancers in 2021

|  | **Global,**  **n(%)** | **East Asia and Pacific-WB,**  **n(%)** | **China,**  **n(%)** | **Japan,**  **n(%)** | **Republic of Korea,**  **n(%)** | **US,**  **n(%)** | **UK,**  **n(%)** |
| --- | --- | --- | --- | --- | --- | --- | --- |
| **Male** |  |  |  |  |  |  |  |
| **Bladder cancer** |  |  |  |  |  |  |  |
| 0-14 years* | 0 (0) | 0 (0) | 0 (0) | 0 (0) | 0 (0) | 0 (0) | 0 (0) |
| 15-49 years | 4522.60 (2.7) | 1755.39 (3.3) | 1285.86 (3.6) | 46.03 (0.6) | 19.66 (1.3) | 157.81 (1.0) | 43.46 (0.9) |
| 50-74 years | 70119.07 (42.6) | 21918.89 (41.6) | 15683.45 (44.4) | 1614.90 (21.4) | 454.74 (31.2) | 5552.71 (36.0) | 1191.94 (25.9) |
| ≥75 years | 89982.24 (54.7) | 29051.30 (55.1) | 18381.08 (52.0) | 5870.98 (77.9) | 982.75 (67.4) | 9721.64 (63.0) | 3375.37 (73.2) |
| Total | 164623.90 (100.0) | 52725.58 (100.0) | 35350.38 (100.0) | 7531.90 (100.0) | 1457.16 (100.0) | 15432.16 (100.0) | 4610.77 (100.0) |
| **Kidney cancer** |  |  |  |  |  |  |  |
| 0-14 years | 1854.85 (1.7) | 319.79 (1.1) | 185.44 (1.1) | 3.31 (0.1) | 1.21 (0.1) | 18.46 (0.2) | 3.67 (0.1) |
| 15-49 years | 7897.71 (7.4) | 2914.93 (9.7) | 2058.38 (11.7) | 99.32 (1.9) | 51.31 (4.9) | 496.21 (4.2) | 125.76 (4.1) |
| 50-74 years | 59478.62 (55.8) | 16071.39 (53.2) | 9839.77 (55.8) | 1880.54 (35.2) | 563.10 (53.3) | 6564.96 (55.3) | 1404.67 (45.7) |
| ≥75 years | 37312.47 (35.0) | 10882.67 (36.0) | 5551.90 (31.5) | 3356.11 (62.9) | 441.08 (41.7) | 4786.86 (40.3) | 1536.31 (50.0) |
| Total | 106543.65 (100.0) | 30188.78 (100.0) | 17635.49 (100.0) | 5339.29 (100.0) | 1056.71 (100.0) | 11866.50 (100.0) | 3070.41 (100.0) |
| **Prostate cancer** |  |  |  |  |  |  |  |
| 0-14 years* | 0 (0) | 0 (0) | 0 (0) | 0 (0) | 0 (0) | 0 (0) | 0 (0) |
| 15-49 years | 2860.93 (0.7) | 744.68 (0.8) | 378.13 (1.0) | 18.67 (0.1) | 7.22 (0.3) | 128.44 (0.3) | 23.61 (0.2) |
| 50-74 years | 156747.97 (36.2) | 30048.09 (34.1) | 12867.72 (34.4) | 2829.73 (17.7) | 670.98 (25.8) | 14351.94 (32.6) | 3177.03 (21.2) |
| ≥75 years | 272854.43 (63.1) | 57341.00 (65.1) | 24117.63 (64.5) | 13143.32 (82.2) | 1923.76 (73.9) | 29551.98 (67.1) | 11820.10 (78.7) |
| Total | 432463.33 (100.0) | 88133.78 (100.0) | 37363.47 (100.0) | 15991.72 (100.0) | 2601.96 (100.0) | 44032.37 (100.0) | 15020.75 (100.0) |
| **Testicular cancer** |  |  |  |  |  |  |  |
| 0-14 years* | 0 (0) | 0 (0) | 0 (0) | 0 (0) | 0 (0) | 0 (0) | 0 (0) |
| 15-49 years | 7391.29 (64.9) | 1049.38 (46.4) | 440.74 (35.4) | 44.91 (41.9) | 7.86 (58.9) | 316.93 (58.3) | 37.16 (46.6) |
| 50-74 years | 2560.39 (22.5) | 729.83 (32.3) | 448.84 (36.1) | 28.89 (27.0) | 3.31 (24.8) | 156.16 (28.7) | 21.32 (26.8) |
| ≥75 years | 1436.62 (12.6) | 483.33 (21.4) | 354.99 (28.5) | 33.38 (31.1) | 2.17 (16.3) | 70.46 (13.0) | 21.20 (26.6) |
| Total | 11388.30 (100.0) | 2262.53 (100.0) | 1244.57 (100.0) | 107.17 (100.0) | 13.33 (100.0) | 543.56 (100.0) | 79.69 (100.0) |
| **Female** |  |  |  |  |  |  |  |
| **Bladder cancer** |  |  |  |  |  |  |  |
| 0-14 years* | 0 (0) | 0 (0) | 0 (0) | 0 (0) | 0 (0) | 0 (0) | 0 (0) |
| 15-49 years | 1805.19 (3.2) | 459.46 (2.7) | 289.51 (3.0) | 22.05 (0.6) | 6.26 (1.2) | 94.14 (1.6) | 30.86 (1.4) |
| 50-74 years | 19955.45 (34.8) | 5639.51 (32.7) | 3940.07 (40.4) | 434.21 (11.0) | 87.01 (17.0) | 1814.19 (29.9) | 509.79 (22.4) |
| ≥75 years | 35503.78 (62.0) | 11151.12 (64.6) | 5533.75 (56.7) | 3482.33 (88.4) | 417.14 (81.7) | 4156.66 (68.5) | 1733.63 (76.2) |
| Total | 57264.42 (100.0) | 17250.10 (100.0) | 9763.33 (100.0) | 3938.58 (100.0) | 510.41 (100.0) | 6064.98 (100.0) | 2274.28 (100.0) |
| **Kidney cancer** |  |  |  |  |  |  |  |
| 0-14 years | 1207.83 (2.2) | 204.13 (1.5) | 104.78 (1.4) | 3.51 (0.1) | 0.89 (0.2) | 21.83 (0.4) | 5.53 (0.3) |
| 15-49 years | 3079.88 (5.6) | 879.89 (6.4) | 467.24 (6.5) | 43.12 (1.4) | 17.74 (4.0) | 202.03 (3.3) | 59.99 (3.1) |
| 50-74 years | 25135.41 (46.0) | 6237.11 (45.1) | 3925.57 (54.3) | 590.32 (19.6) | 149.99 (33.7) | 2728.97 (44.3) | 692.85 (35.9) |
| ≥75 years | 25227.77 (46.2) | 6504.80 (47.0) | 2734.23 (37.8) | 2380.26 (78.9) | 276.64 (62.1) | 3206.79 (52.1) | 1173.02 (60.7) |
| Total | 54650.89 (100.0) | 13825.93 (100.0) | 7231.82 (100.0) | 3017.20 (100.0) | 445.26 (100.0) | 6159.62 (100.0) | 1931.39 (100.0) |

*Deaths data provide in GBD 2021 for bladder cancer, prostate cancer, and testicular cancer in aged 0-14 are 0.

# Table S3. The **male to female ratios of incidence and mortality in** genitourinary cancers varies **by age, regions and countries in 2021**

|  | **Male to female ratios of incidence** | | | | **Male to female ratios of mortality** | | | | |
| --- | --- | --- | --- | --- | --- | --- | --- | --- | --- |
|  | **0-14 years*** | **15-49 years** | **50-74 years** | **≥75 years** |  | **0-14 years*** | **15-49 years** | **50-74 years** | **≥75 years** |
| **Bladder cancer** |  |  |  |  |  |  |  |  |  |
| Global | - | 2.94 | 4.15 | 3.97 |  | - | 2.44 | 3.70 | 3.51 |
| East Asia and Pacific-WB | - | 4.04 | 4.50 | 4.15 |  | - | 3.60 | 3.99 | 3.58 |
| China | - | 4.53 | 4.43 | 4.78 |  | - | 4.11 | 4.00 | 4.32 |
| Japan | - | 2.55 | 4.55 | 3.39 |  | - | 2.01 | 3.86 | 2.54 |
| Republic of Korea | - | 3.50 | 6.35 | 5.00 |  | - | 2.87 | 5.35 | 3.86 |
| US | - | 1.69 | 3.44 | 3.64 |  | - | 1.66 | 3.31 | 3.25 |
| UK | - | 1.66 | 2.81 | 2.98 |  | - | 1.44 | 2.44 | 2.52 |
| **Kidney cancer** |  |  |  |  |  |  |  |  |  |
| Global | 1.00 | 1.90 | 2.20 | 2.05 |  | 1.44 | 2.50 | 2.49 | 2.05 |
| East Asia and Pacific-WB | 1.10 | 2.51 | 2.46 | 2.42 |  | 1.42 | 3.12 | 2.64 | 2.30 |
| China | 1.14 | 3.02 | 2.37 | 2.73 |  | 1.55 | 4.07 | 2.52 | 2.64 |
| Japan | 0.67 | 1.62 | 2.92 | 2.26 |  | 0.89 | 2.22 | 3.30 | 2.12 |
| Republic of Korea | 0.99 | 1.97 | 3.40 | 2.69 |  | 1.31 | 2.64 | 3.85 | 2.61 |
| US | 0.61 | 1.83 | 2.27 | 2.19 |  | 0.81 | 2.44 | 2.60 | 2.07 |
| UK | 0.47 | 1.56 | 1.87 | 1.76 |  | 0.64 | 2.14 | 2.11 | 1.70 |

*Incidence and mortality data provide in GBD 2021 for bladder cancer in aged 0-14 are 0.

# Table S4. Trends in annual ASIRs and ASMRs by across ages and locations, 1990-2021

|  | **AAPCs (95%CI) of ASIR, 1990-2021** | | | |  | **AAPCs (95%CI) of ASMR, 1990-2021** | | | |
| --- | --- | --- | --- | --- | --- | --- | --- | --- | --- |
|  | **0-14 years*** | **15-49 years** | **50-74 years** | **≥75 years** |  | **0-14 years*** | **15-49 years** | **50-74 years** | **≥75 years** |
| **Male** |  |  |  |  |  |  |  |  |  |
| **Bladder cancer** |  |  |  |  |  |  |  |  |  |
| Global | - | 0.41 (0.26 - 0.56) | -0.33 (-0.47 - -0.18) | -0.06 (-0.15 - 0.02) |  | - | -0.75 (-0.86 - -0.64) | -1.18 (-1.28 - -1.07) | -0.45 (-0.57 - -0.33) |
| East Asia and Pacific-WB | - | 1.70 (1.57 - 1.84) | 0.47 (0.32 - 0.62) | 0.77 (0.66 - 0.88) |  | - | -0.34 (-0.47 - -0.21) | -1.08 (-1.26 - -0.90) | -0.06 (-0.20 - 0.07) |
| China | - | 2.14 (1.95 - 2.34) | 0.61 (0.38 - 0.84) | 0.77 (0.55 - 0.99) |  | - | -0.31 (-0.53 - -0.09) | -1.31 (-1.53 - -1.08) | -0.37 (-0.65 - -0.09) |
| Japan | - | 0.58 (-0.01 - 1.17) | 0.74 (0.21 - 1.28) | 0.47 (0.10 - 0.83) |  | - | -0.30 (-0.84 - 0.23) | 0.07 (-0.28 - 0.42) | 0.44 (0.10 - 0.79) |
| Republic of Korea | - | 1.04 (0.71 - 1.36) | 0.04 (-0.20 - 0.28) | 0.84 (0.50 - 1.19) |  | - | -1.54 (-1.94 - -1.15) | -2.30 (-2.54 - -2.05) | -0.62 (-1.01 - -0.23) |
| US | - | 0.33 (-0.10 - 0.75) | -0.21 (-0.54 - 0.13) | 0.32 (0.08 - 0.57) |  | - | -0.39 (-0.93 - 0.15) | -0.81 (-0.96 - -0.66) | 0.30 (-0.04 - 0.63) |
| UK | - | -0.66 (-1.49 - 0.17) | -2.21 (-2.66 - -1.75) | -0.73 (-0.84 - -0.62) |  | - | -1.37 (-2.31 - -0.42) | -2.77 (-3.19 - -2.39) | -0.90 (-1.27 - -0.54) |
| **Kidney cancer** |  |  |  |  |  |  |  |  |  |
| Global | -0.19 (-0.44 - 0.07) | 1.34 (1.21 - 1.47) | 0.63 (0.56 - 0.70) | 0.89 (0.80 - 0.97) |  | -0.81 (-0.99 - -0.63) | 0.38 (0.22 - 0.54) | -0.19 (-0.33 - -0.05) | 0.54 (0.47 - 0.61) |
| East Asia and Pacific-WB | -0.66 (-1.04 - -0.28) | 3.69 (3.43 - 3.96) | 2.06 (1.90 - 2.22) | 2.07 (1.99 - 2.15) |  | -2.58 (-2.94 - -2.22) | 1.78 (1.61 - 1.96) | 0.62 (0.41 - 0.83) | 1.4 (1.28 - 1.52) |
| China | -0.98 (-1.55 - -0.41) | 4.48 (4.22 - 4.73) | 2.75 (2.47 - 3.02) | 2.14 (1.80 - 2.49) |  | -3.33 (-4.24 - -2.42) | 2.13 (1.88 - 2.38) | 0.87 (0.45 - 1.29) | 1.11 (0.66 - 1.56) |
| Japan | -0.32 (-1.37 - 0.75) | 1.19 (0.74 - 1.65) | 1.13 (0.54 - 1.72) | 1.62 (1.26 - 1.99) |  | -2.06 (-3.15 - -0.95) | -0.32 (-1.20 - 0.56) | 0.19 (-0.26 - 0.63) | 1.54 (1.13 - 1.96) |
| Republic of Korea | -0.09 (-0.6 - 0.43) | 3.72 (3.41 - 4.03) | 2.39 (1.92 - 2.85) | 3.44 (2.93 - 3.95) |  | -3.23 (-3.96 - -2.49) | 0.82 (0.57 - 1.08) | 0.42 (0.22 - 0.61) | 2.24 (1.95 - 2.54) |
| US | -0.75 (-1.05 - -0.45) | 0 (-0.50 - 0.50) | -0.04 (-0.51 - 0.43) | 0.81 (0.52 - 1.10) |  | -1.87 (-2.44 - -1.30) | -0.89 (-1.11 - -0.67) | -0.86 (-1.25 - -0.46) | 0.25 (0.04 - 0.59) |
| UK | -0.68 (-1.85 - 0.5) | 1.48 (0.93 - 2.03) | 0.69 (0.21 - 1.18) | 1.71 (1.17 - 2.25) |  | -1.97 (-3.28 - -0.64) | 0.16 (-0.43 - 0.75) | -0.43 (-0.71 - -0.15) | 1.24 (0.89 - 1.59) |
| **Prostate cancer** |  |  |  |  |  |  |  |  |  |
| Global | - | 2.08 (1.84 - 2.32) | 0.83 (0.72 - 0.93) | -0.36 (-0.46 - -0.26) |  | - | 0.43 (0.32 - 0.55) | -0.52 (-0.58 - -0.46) | -0.64 (-0.76 - -0.53) |
| East Asia and Pacific-WB | - | 3.89 (3.73 - 4.05) | 2.52 (2.19 - 2.86) | 1.21 (0.99 - 1.42) |  | - | 0.86 (0.75 - 0.98) | 0.17 (0.02 - 0.32) | 0.46 (0.27 - 0.66) |
| China | - | 4.11 (3.97 - 4.25) | 3.26 (3.01 - 3.52) | 1.92 (1.70 - 2.14) |  | - | 0.35 (0.19 - 0.50) | 0.03 (-0.23 - 0.28) | 0.79 (0.45 - 1.13) |
| Japan | - | 3.76 (2.60 - 4.93) | 4.15 (3.83 - 4.46) | 1.67 (1.21 - 2.14) |  | - | 0.28 (-0.16 - 0.71) | 1.01 (0.558 - 1.47) | 0.85 (0.58 - 1.12) |
| Republic of Korea | - | 4.75 (4.30 - 5.20) | 4.92 (4.18 - 5.66) | 4.24 (3.98 - 4.50) |  | - | -0.06 (-0.52 - 0.40) | 0.35 (0.07 - 0.63) | 1.54 (1.38 - 1.70) |
| US | - | 1.79 (1.23 - 2.34) | -0.52 (-0.83 - -0.20) | -1.45 (-1.66 - -1.24) |  | - | -0.05 (-0.62 - 0.53) | -2.16 (-2.45 - -1.88) | -1.60 (-1.75 - -1.44) |
| UK | - | 2.41 (1.80 - 3.02) | 1.07 (0.85 - 1.30) | 0.18 (-0.05 - 0.40) |  | - | -0.35 (-0.96 - 0.26) | -1.74 (-2.00 - -1.47) | -0.41 (-0.64 - -0.19) |
| **Testicular cancer** |  |  |  |  |  |  |  |  |  |
| Global | - | 1.43 (1.01 - 1.85) | 1.19 (0.91 - 1.46) | 0.13 (-0.08 - 0.34) |  | - | -0.26 (-0.42 - -0.11) | -0.70 (-0.84 - -0.57) | -0.51 (-0.76 - -0.27) |
| East Asia and Pacific-WB | - | 2.01 (1.42 - 2.60) | 1.9 (1.27 - 2.53) | 1.22 (0.64 - 1.81) |  | - | -0.57 (-0.73 - -0.41) | -0.66 (-1.05 - -0.26) | 0.08 (-0.24 - 0.41) |
| China | - | 3.76 (3.08 - 4.45) | 2.73 (2.12 - 3.34) | 1.43 (0.92 - 1.95) |  | - | -1.40 (-1.84 - -0.96) | -1.08 (-1.54 - -0.62) | 0.07 (-0.65 - 0.79) |
| Japan | - | 0.67 (-0.52 - 1.86) | 1.49 (-0.43 - 3.45) | -0.46 (-2.17 - 1.28) |  | - | -1.94 (-2.95 - -0.92) | -1.00 (-2.88 - 0.92) | -1.29 (-2.18 - -0.40) |
| Republic of Korea | - | 4.11 (3.79 - 4.44) | 2.49 (2.10 - 2.88) | 0.42 (0.13 - 0.71) |  | - | -2.72 (-2.89 - -2.55) | -3.07 (-3.27 - -2.86) | -2.17 (-2.49 - -1.84) |
| US | - | 1.21 (0.30 - 2.13) | 1.24 (0.94 - 1.54) | -0.1 (-1.38 - 1.20) |  | - | -0.50 (-0.75 - -0.25) | -0.10 (-0.21 - 0.02) | -0.42 (-1.68 - 0.86) |
| UK | - | -1.10 (-1.68 - -0.52) | -1.42 (-1.88 - -0.96) | -3.22 (-3.59 - -2.84) |  | - | -3.43 (-3.79 - -3.08) | -3.92 (-4.23 - -3.60) | -3.37 (-3.72 - -3.03) |
| **Female** |  |  |  |  |  |  |  |  |  |
| **Bladder cancer** |  |  |  |  |  |  |  |  |  |
| Global | - | 0.20 (0.02 - 0.37) | -0.50 (-0.63 - -0.36) | -0.34 (-0.45 - -0.23) |  | - | -0.77 (-0.87 - -0.66) | -1.31 (-1.40 - -1.23) | -0.56 (-0.69 - -0.43) |
| East Asia and Pacific-WB | - | 0.51 (0.32 - 0.71) | -0.58 (-0.71 - -0.44) | 0 (-0.21 - 0.21) |  | - | -1.50 (-1.62 - -1.38) | -2.13 (-2.26 - -2.00) | -0.59 (-0.78 - -0.41) |
| China | - | 0.39 (0.11 - 0.68) | -0.70 (-0.89 - -0.52) | -0.51 (-0.95 - -0.07) |  | - | -1.89 (-2.03 - -1.75) | -2.57 (-2.73 - -2.41) | -1.43 (-1.84 - -1.01) |
| Japan | - | 1.94 (1.44 - 2.45) | 0.52 (-0.06 - 1.10) | 0.70 (0.33 - 1.06) |  | - | 1.02 (0.60 - 1.45) | -0.25 (-0.70 - 0.20) | 0.92 (0.74 - 1.09) |
| Republic of Korea | - | 1.86 (1.55 - 2.18) | -0.73 (-1.13 - -0.33) | 0.69 (0.38 - 0.99) |  | - | -0.58 (-0.91 - -0.25) | -2.86 (-3.14 - -2.58) | -0.41 (-0.73 - -0.09) |
| US | - | 0.88 (0.59 - 1.17) | -0.37 (-0.52 - -0.21) | 0.23 (0.08 - 0.37) |  | - | 0.26 (-0.03 - 0.55) | -0.90 (-1.08 - -0.73) | 0.12 (0.02 - 0.23) |
| UK | - | 0.13 (-0.35 - 0.62) | -1.35 (-1.55 - -1.16) | 0.04 (-0.21 - 0.29) |  | - | -0.46 (-0.74 - -0.17) | -1.91 (-2.13 - -1.68) | 0.02 (-0.26 - 0.30) |
| **Kidney cancer** |  |  |  |  |  |  |  |  |  |
| Global | -1.37 (-1.58 - -1.15) | 0.95 (0.70 - 1.20) | 0.02 (-0.10 - 0.14) | 0.48 (0.39 - 0.56) |  | -2.28 (-2.43 - -2.13) | -0.22 (-0.45 - 0.02) | -0.80 (-1.00 - -0.61) | 0.21 (0.09 - 0.33) |
| East Asia and Pacific-WB | -2.20 (-2.83 - -1.57) | 2.77 (2.57 - 2.97) | 1.59 (1.41 - 1.78) | 1.65 (1.52 - 1.79) |  | -4.19 (-4.72 - -3.66) | 0.77 (0.55 - 1.00) | -0.02 (-0.23 - 0.18) | 1.08 (1.01 - 1.15) |
| China | -2.73 (-3.68 - -1.78) | 3.08 (2.83 - 3.33) | 1.95 (1.61 - 2.30) | 1.37 (0.88 - 1.86) |  | -5.30 (-6.27 - -4.29) | 0.37 (0.12 - 0.63) | 0 (-0.31 - 0.30) | 0.34 (-0.14 - 0.83) |
| Japan | 0.87 (0.43 - 1.32) | 1.94 (1.02 - 2.88) | 1.07 (0.12 - 2.04) | 1.83 (1.47 - 2.19) |  | -0.89 (-1.18 - -0.60) | 0.17 (-0.65 - 1.00) | -0.08 (-0.53 - 0.37) | 1.93 (1.57 - 2.30) |
| Republic of Korea | -0.15 (-0.69 - 0.38) | 3.74 (3.44 - 4.05) | 1.8 (1.36 - 2.24) | 3.24 (2.76 - 3.72) |  | -3.57 (-4.26 - -2.87) | 0.44 (-0.01 - 0.90) | -0.50 (-0.83 - -0.16) | 2.26 (1.92 - 2.60) |
| US | -0.59 (-1.50 - 0.33) | -0.43 (-1.23 - 0.38) | -0.54 (-0.73 - -0.35) | 0.61 (0.45 - 0.78) |  | -1.49 (-2.28 - -0.70) | -1.43 (-2.28 - -0.57) | -1.37 (-1.61 - -1.12) | 0.30 (0.14 - 0.47) |
| UK | -0.26 (-1.15 - 0.65) | 1.66 (1.25 - 2.07) | 0.56 (-0.01 - 1.14) | 2.22 (1.97 - 2.47) |  | -0.93 (-1.40 - -0.45) | 0.29 (-0.20 - 0.78) | -0.56 (-1.09 - -0.03) | 1.74 (1.52 - 1.97) |

*Incidence and mortality data provide in GBD 2021 for bladder cancer, prostate cancer, and testicular cancer in aged 0-14 are 0.

AAPCs, average annual percent changes; ASIRs, age-standardized incidence rates; ASMRs, age-standardized mortality rate; CI, confidence interval

# Table S5. **Decomposition analysis on changes in** genitourinary cancers **incidence** across sexes and locations from 1990 to 2021

|  | **Overall difference** | **Absolute incidence changes due to individual factors (contribution proportions to the overall changes, %)** | | |
| --- | --- | --- | --- | --- |
|  |  | **Aging** | **Population growth** | **Epidemiological change** |
| **Male** |  |  |  |  |
| **Bladder cancer** |  |  |  |  |
| Global | 221832 | 95812 (43.19%) | 142874 (64.41%) | -16855 (-7.60%) |
| East Asia and Pacific-WB | 88453 | 42873 (48.47%) | 28303 (32.00%) | 17277 (19.53%) |
| China | 59062 | 30593 (51.80%) | 14935 (25.29%) | 13533 (22.91%) |
| Japan | 12766 | 8812 (69.03%) | 1153 (9.03%) | 2801 (21.94%) |
| Republic of Korea | 3707 | 2406 (64.90%) | 915 (24.68%) | 386 (10.42%) |
| US | 32157 | 16445 (51.14%) | 15525 (48.28%) | 187 (0.58%) |
| UK | 251 | 2800 (1115.72%) | 1986 (791.33%) | -4535 (-1807.05%) |
| **Kidney cancer** |  |  |  |  |
| Global | 156239 | 54872 (35.12%) | 62681 (40.12%) | 38686 (24.76%) |
| East Asia and Pacific-WB | 56024 | 19238 (34.34%) | 9796 (17.49%) | 26990 (48.18%) |
| China | 36578 | 11502 (31.45%) | 4613 (12.61%) | 20463 (55.94%) |
| Japan | 6371 | 3556 (55.81%) | 39 (0.61%) | 2776 (43.58%) |
| Republic of Korea | 2189 | 985 (44.97%) | 196 (8.96%) | 1009 (46.08%) |
| US | 20832 | 10763 (51.67%) | 8458 (40.60%) | 1611 (7.73%) |
| UK | 3465 | 1173 (33.85%) | 858 (24.76%) | 1434 (41.40%) |
| **Prostate cancer** |  |  |  |  |
| Global | 817978 | 299672 (36.64%) | 414003 (50.61%) | 104302 (12.75%) |
| East Asia and Pacific-WB | 184644 | 73968 (40.06%) | 42197 (22.85%) | 68478 (37.09%) |
| China | 74847 | 29069 (38.84%) | 12516 (16.72%) | 33262 (44.44%) |
| Japan | 44022 | 19761 (44.89%) | 2396 (5.44%) | 21865 (49.67%) |
| Republic of Korea | 9784 | 3793 (38.77%) | 1351 (13.81%) | 4639 (47.42%) |
| US | 111283 | 88283 (79.33%) | 80123 (72.00%) | -57123 (-51.33%) |
| UK | 24497 | 10094 (41.21%) | 7000 (28.58%) | 7402 (30.22%) |
| **Testicular cancer** |  |  |  |  |
| Global | 52674 | -3335 (-6.33%) | 30058 (57.06%) | 25950 (49.27%) |
| East Asia and Pacific-WB | 8409 | -701 (-8.33%) | 3452 (41.06%) | 5657 (67.27%) |
| China | 4856 | -29 (-0.59%) | 1173 (24.14%) | 3713 (76.45%) |
| Japan | 136 | -547 (-403.17%) | 192 (141.44%) | 491 (361.74%) |
| Republic of Korea | 273 | -68 (-25.02%) | 80 (29.27%) | 262 (95.75%) |
| US | 4896 | -1247 (-25.47%) | 3066 (62.63%) | 3076 (62.84%) |
| UK | -560 | -205 (-36.67%) | 462 (82.52%) | -817 (-145.85%) |
| **Female** |  |  |  |  |
| **Bladder cancer** |  |  |  |  |
| Global | 58336 | 25034 (42.91%) | 44853 (76.89%) | -11550 (-19.80%) |
| East Asia and Pacific-WB | 19791 | 13067 (66.03%) | 8798 (44.46%) | -2074 (-10.48%) |
| China | 10916 | 8905 (81.58%) | 4674 (42.82%) | -2664 (-24.40%) |
| Japan | 4664 | 3204 (68.69%) | 472 (10.11%) | 989 (21.20%) |
| Republic of Korea | 849 | 567 (66.78%) | 223 (26.28%) | 59 (6.94%) |
| US | 9104 | 3779 (41.51%) | 5483 (60.22%) | -157 (-1.73%) |
| UK | 325 | 341 (105.11%) | 678 (208.87%) | -695 (-213.98%) |
| **Kidney cancer** |  |  |  |  |
| Global | 71816 | 28042 (39.05%) | 37295 (51.93%) | 6478 (9.02%) |
| East Asia and Pacific-WB | 22484 | 8821 (39.23%) | 4903 (21.81%) | 8760 (38.96%) |
| China | 12989 | 5196 (40.00%) | 2314 (17.82%) | 5479 (42.18%) |
| Japan | 3340 | 1738 (52.04%) | 78 (2.34%) | 1524 (45.62%) |
| Republic of Korea | 834 | 356 (42.67%) | 83 (10.00%) | 395 (47.33%) |
| US | 8410 | 4356 (51.80%) | 4686 (55.72%) | -632 (-7.52%) |
| UK | 2050 | 333 (16.23%) | 502 (24.48%) | 1215 (59.29%) |

# Table S6. **Decomposition analysis on changes in** genitourinary cancers **mortality** across sexes and locations from 1990 to 2021

|  | **Overall changes** | **Absolute mortality changes due to individual factors (contribution proportions)** | | |
| --- | --- | --- | --- | --- |
|  |  | **Aging** | **Population growth** | **Epidemiological change** |
| **Male** |  |  |  |  |
| **Bladder cancer** |  |  |  |  |
| Global | 75310 | 46113 (61.23%) | 60733 (80.64%) | -31536 (-41.87%) |
| East Asia and Pacific-WB | 30007 | 23029 (76.75%) | 13017 (43.38%) | -6039 (-20.13%) |
| China | 19136 | 18138 (94.78%) | 7404 (38.69%) | -6405 (-33.47%) |
| Japan | 4864 | 3904 (80.26%) | 402 (8.27%) | 558 (11.47%) |
| Republic of Korea | 937 | 1028 (109.74%) | 329 (35.09%) | -420 (-44.83%) |
| US | 7500 | 4216 (56.21%) | 3754 (50.05%) | -470 (-6.27%) |
| UK | 291 | 1534 (526.60%) | 933 (320.28%) | -2176 (-746.88%) |
| **Kidney cancer** |  |  |  |  |
| Global | 58645 | 28464 (48.54%) | 28401 (48.43%) | 1780 (3.04%) |
| East Asia and Pacific-WB | 20406 | 10834 (53.09%) | 4403 (21.58%) | 5169 (25.33%) |
| China | 11890 | 6605 (55.55%) | 1979 (16.64%) | 3306 (27.81%) |
| Japan | 3354 | 2367 (70.58%) | 20 (0.60%) | 967 (28.82%) |
| Republic of Korea | 814 | 545 (66.94%) | 89 (11.00%) | 179 (22.06%) |
| US | 4980 | 3614 (72.57%) | 2539 (50.98%) | -1173 (-23.55%) |
| UK | 1272 | 685 (53.82%) | 425 (33.42%) | 162 (12.77%) |
| **Prostate cancer** |  |  |  |  |
| Global | 220492 | 127548 (57.85%) | 152281 (69.06%) | -59337 (-26.91%) |
| East Asia and Pacific-WB | 61474 | 36947 (60.10%) | 18784 (30.56%) | 5743 (9.34%) |
| China | 26680 | 17329 (64.95%) | 6398 (23.98%) | 2953 (11.07%) |
| Japan | 11603 | 8006 (69.00%) | 782 (6.74%) | 2814 (24.26%) |
| Republic of Korea | 2209 | 1359 (61.49%) | 418 (18.94%) | 433 (19.58%) |
| US | 6684 | 16194 (242.29%) | 14076 (210.61%) | -23586 (-352.90%) |
| UK | 4223 | 4666 (110.48%) | 2634 (62.38%) | -3077 (-72.86%) |
| **Testicular cancer** |  |  |  |  |
| Global | 3770 | 306 (8.10%) | 4612 (122.34%) | -1148 (-30.45%) |
| East Asia and Pacific-WB | 610 | 263 (43.11%) | 698 (114.39%) | -351 (-57.50%) |
| China | 197 | 257 (130.17%) | 335 (170.14%) | -395 (-200.31%) |
| Japan | -38 | 10 (26.52%) | 10 (27.00%) | -59 (-153.53%) |
| Republic of Korea | -10 | 0.1 (1.52%) | 6 (66.84%) | -16 (-165.32%) |
| US | 90 | -6 (-6.94%) | 166 (183.11%) | -69 (-76.17%) |
| UK | -101 | 9 (8.62%) | 28 (27.77%) | -138 (-136.38%) |
| **Female** |  |  |  |  |
| **Bladder cancer** |  |  |  |  |
| Global | 23453 | 13508 (57.60%) | 22312 (95.14%) | -12368 (-52.73%) |
| East Asia and Pacific-WB | 7849 | 8338 (106.24%) | 4928 (62.78%) | -5417 (-69.02%) |
| China | 3212 | 5996 (186.66%) | 2738 (85.23%) | -5522 (-171.90%) |
| Japan | 2682 | 1937 (72.23%) | 233 (8.70%) | 511 (19.07%) |
| Republic of Korea | 318 | 324 (101.96%) | 109 (34.36%) | -115 (-36.32%) |
| US | 2183 | 947 (43.35%) | 1521 (69.67%) | -284 (-13.02%) |
| UK | 188 | 175 (93.45%) | 387 (206.07%) | -374 (-199.52%) |
| **Kidney cancer** |  |  |  |  |
| Global | 25129 | 14182 (56.44%) | 16147 (64.26%) | -5201 (-20.70%) |
| East Asia and Pacific-WB | 8345 | 5330 (63.87%) | 2230 (26.72%) | 785 (9.40%) |
| China | 3926 | 3125 (79.60%) | 1000 (25.48%) | -199 (-5.08%) |
| Japan | 2063 | 1315 (63.75%) | 43 (2.10%) | 704 (34.14%) |
| Republic of Korea | 327 | 221 (67.82%) | 39 (12.08%) | 66 (20.10%) |
| US | 1851 | 1340 (72.42%) | 1375 (74.30%) | -865 (-46.72%) |
| UK | 736 | 164 (22.22%) | 245 (33.27%) | 328 (44.50%) |

# Table S7. Relative risks of smoking, high BMI, and high FPG **for genitourinary cancers**

|  | **Relative risk (95% UI)** | | |
| --- | --- | --- | --- |
|  | **Bladder cancer** | **Kidney cancer** | **Prostate cancer** |
| **High FPG (above 4.8-5.4 mmol/L)** | Male:  1.51 (1.08 to 2.26) Female:  1.51 (1.08 to 2.25) | - | - |
| **High BMI (greater than 20 to 25 kg/m2)** | - | Male:  1.24 (1.17 to 1.31) Female:  1.32 (1.25 to 1.39) | - |
| **Smoking (Both sexes)** |  |  |  |
| 10 Pack Years | 2.20 (1.57 to 2.88) | 1.30 (1.07 to 1.55) | 1.19 (1.03 to 1.35) |
| 20 Pack Years | 2.98 (1.99 to 4.09) | 1.59 (1.28 to 1.92) | 1.17 (1.02 to 1.33) |
| 30 Pack Years | 3.33 (2.27 to 4.61) | 1.74 (1.38 to 2.13) | 1.23 (1.03 to 1.47) |
| 40 Pack Years | 3.94 (2.63 to 5.51) | 1.79 (1.40 to 2.24) | 1.35 (0.96 to 1.79) |
| 50 Pack Years | 4.34 (2.81 to 6.21) | 1.90 (1.49 to 2.35) | - |
| 60 Pack Years | 4.55 (2.82 to 6.72) | 1.99 (1.38 to 2.73) | - |
| 70 Pack Years | 4.74 (2.44 to 7.96) | - |  |

BMI, body mass index; FPG, fasting plasma glucose; RR, relative risk; UI, uncertainty interval
