# Supplementary material for: Incidence, mortality, and risk factors of bladder, kidney, prostate and testicular cancers in China and comparisons with the United States, the United Kingdom, Japan, and the Republic of Korea: an up-to-date overview based on the Global Burden of Disease 2021
Source: Exp Hematol Oncol. 2025 Aug 6;14:103. doi: 10.1186/s40164-025-00694-9 (PMC12329898; doi:10.1186/s40164-025-00694-9)
Supplement: Supplementary file 3 — Supplementary Material 3 [file 40164_2025_694_MOESM3_ESM.docx]

# Supplementary figures

**Figure S1.** **Attributable numbers of genitourinary cancer mortality due to smoking across sexes, locations, and ages from 1990 to 2021**

Attributable numbers of genitourinary cancer (kidney, bladder, and prostate cancers) mortality due to smoking in **(A)** 15-49 years, **(B)** 50-74 years, and **(C)** ≥75 years age groups across sexes (male and female) and geographic locations (global, East Asia and Pacific, China, Japan, the Republic of Korea, the US and the UK) from 1990 to 2021.

**Figure S2.** **Attributable numbers of bladder cancer mortality due to high fasting plasma glucose across sexes, locations, and ages from 1990 to 2021**

Attributable numbers of bladder cancer mortality due to high fasting plasma glucose across sexes (male and female) , age groups (15-49 years, 50-74 years and ≥75 years), and geographic locations (global, East Asia and Pacific, China, Japan, the Republic of Korea, the US and the UK) from 1990 to 2021.

**Figure S3. Attributable numbers of kidney cancer mortality due to high body-mass index across sexes, locations, and ages from 1990 to 2021**

Attributable numbers of kidney cancer mortality due to high body-mass index across sexes (male and female) , age groups (15-49 years, 50-74 years and ≥75 years), and geographic locations (global, East Asia and Pacific, China, Japan, the Republic of Korea, the US and the UK) from 1990 to 2021.
