# Supplementary figures and images for: Incidence, mortality, and risk factors of bladder, kidney, prostate and testicular cancers in China and comparisons with the United States, the United Kingdom, Japan, and the Republic of Korea: an up-to-date overview based on the Global Burden of Disease 2021
Source: Exp Hematol Oncol. 2025 Aug 6;14:103. doi: 10.1186/s40164-025-00694-9 (PMC12329898; doi:10.1186/s40164-025-00694-9)

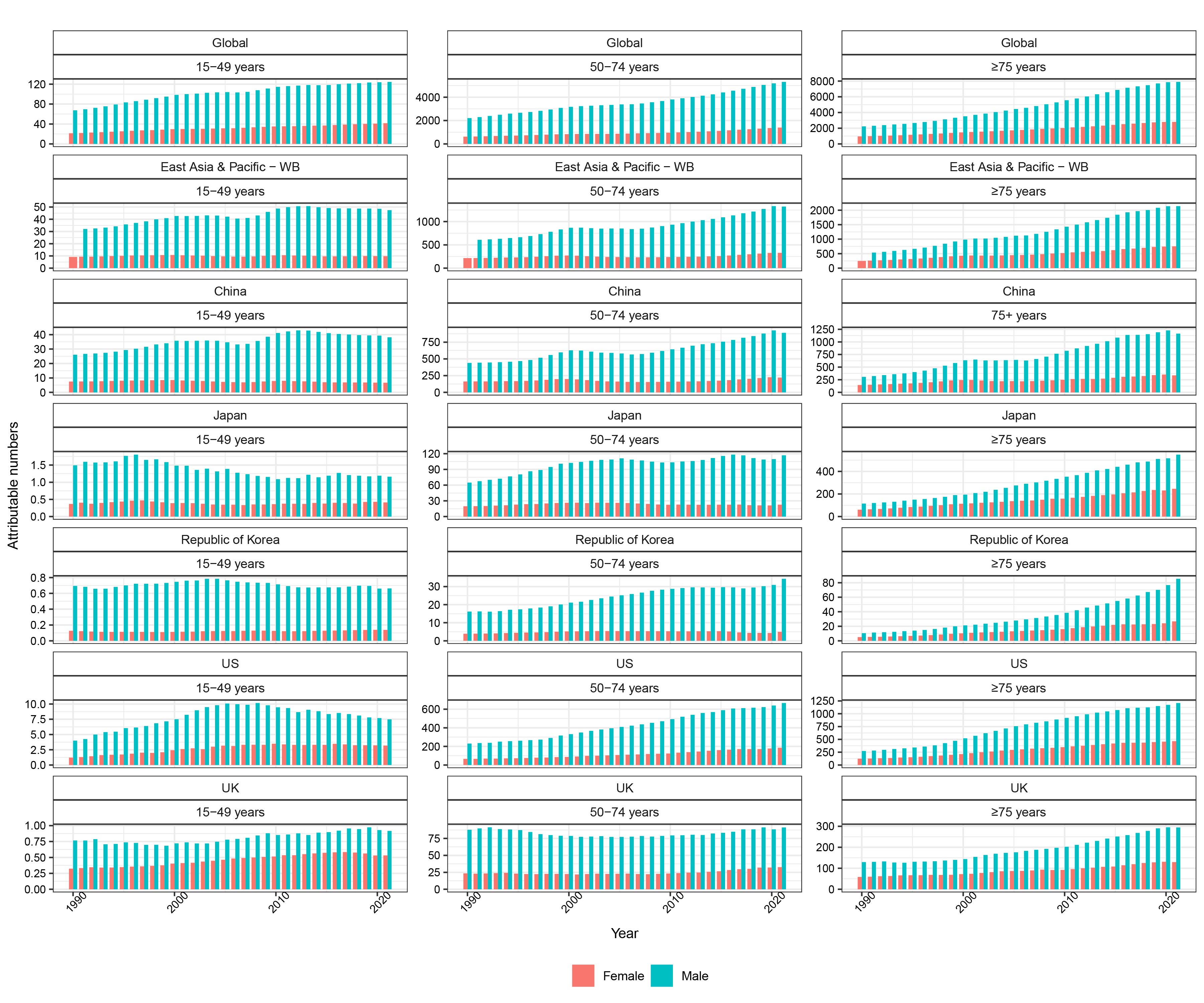

Supplement: Supplementary file 5 — Supplementary Material 5 [file 40164_2025_694_MOESM5_ESM.jpg]

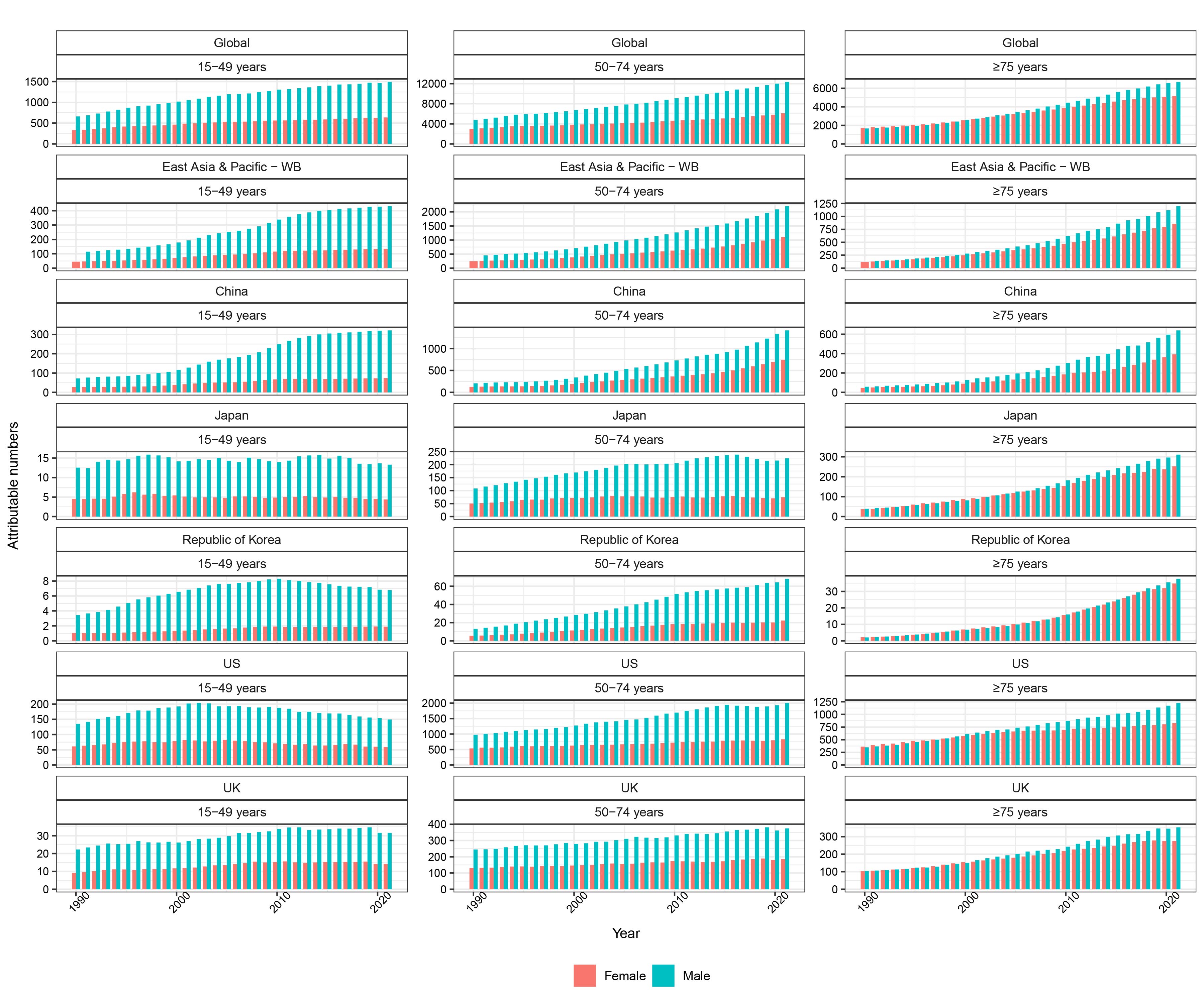

Supplement: Supplementary file 6 — Supplementary Material 6 [file 40164_2025_694_MOESM6_ESM.jpg]
